# Exploring stationary phase morphologies as pathways to greener and faster LC for analyzing sofosbuvir and ledipasvir

**DOI:** 10.1038/s41598-025-31605-x

**Published:** 2025-12-15

**Authors:** Adel Ehab Ibrahim, Samy G. Alamir, Baher I. Salman, Sami El Deeb, Ahmed Al-Harrasi

**Affiliations:** 1 Natural and Medical Sciences Research Center, University of Nizwa, P.O. Box 33, Birkat Al Mauz, Nizwa, 616 Sultanate of Oman; 2 Pharmaceutical analytical chemistry department, Faculty of Pharmacy, Port-Said University, Port-Said, 42511 Egypt; 3 Pharmaceutical Analytical Chemistry Department, Faculty of Pharmacy, Ain Shams University, Abassia, 11566 Cairo Egypt; 4 Pharmaceutical Analytical Chemistry Department, Faculty of Pharmacy, Al-Azhar University, Assiut branch, Assiut, 71524 Egypt; 5 Institute of Medicinal and Pharmaceutical Chemistry, Technische Universitaet Braunschweig, 38106 Braunschweig, Germany

**Keywords:** Green chromatography, Core-shell particles, Totally porous particles, Monolithic column, Ledipasvir, Sofosbuvir, Chemistry, Environmental sciences

## Abstract

Green chemistry has a beneficial influence on the environment and the pharmaceutical enterprises’ economy. Research and quality control laboratories worldwide conduct millions of analyses daily, employing liquid chromatography as the primary analytical technique. Furthermore, advancements in stationary phase technologies aimed at achieving faster and more efficient separations have introduced a new objective for chromatographers. This objective is to supersede conventional methodologies that rely heavily on substantial quantities of organic solvents, pose environmental hazards and incur considerable economic costs. The aim of this work is to provide a brief comparison between monolithic, core-shell and traditional fully porous stationary phases. This could help analysts by offering options for high-speed versus high-resolution separation when choosing the best packing materials. The analytes used in the study are two essential drugs used in the treatment of hepatitis C virus: Sofosbuvir and Ledipasvir, which are co-formulated in tablet dosage forms. The monolithic column enabled the fastest analysis, whereas the core-shell provided the highest efficiency. Validation was conducted in accordance with ICH guidelines using the RP-C18 monolithic column, a mobile phase consisting of 0.1% acetic acid and ethanol (60:40), a flow rate of 1 mL.min^− 1^, and detection at 210 nm. The total analysis time did not exceed 3 min, achieving high accuracy and sensitivity while minimizing the use of hazardous solvents. It achieved a very good score on different green analytical chemistry assessment metrics; 0.7 on AGREE, 76 on MoGAPI, 80 on BAGI, and 73 on CACI.

**Supplementary Information:**

The online version contains supplementary material available at 10.1038/s41598-025-31605-x.

## Introduction

In contemporary times, the importance of time is paramount across multiple sectors, including academic research, industry, and pharmaceuticals. Consequently, significant efforts are devoted to the development of high-throughput methodologies that can expedite research and quality control processes. Additionally, reducing analysis time in is crucial for minimizing economic losses, especially for industrial processes, as it improves quality control, minimizes energy consumption, and could minimize loss of valuable reagents if one considers the flow rate and waste generated. Quick analysis allows pharmaceutical industry to respond promptly to production issues, and facilitates real-time monitoring, which helps also to reduce waste. All this led to high-performance liquid chromatography (HPLC) emerging as one of the preferred techniques for various scientific and industrial applications^1^. Notably, the term “fast HPLC” is a relative concept and lacks a precise definition; It is considered a suboptimal measure to assess chromatographic performance, as another crucial parameter exists: the number of separated analytes per unit time.

Currently, the primary strategy adopted to achieve rapid liquid chromatographic analysis involves altering the morphology of the packed materials within chromatographic columns. Despite their high efficiency in chromatographic separation, sub-2-µm particles require specialized instrumentation to mitigate the high back pressure generated and peak broadening when different mobile phases flow through them^2^. This led to the development of core-shell and monolithic columns. Monolithic silica rods dates back to the 1990s, where they were first introduced by Tanaka group and were then developed by Nakanishi^3,4^.Meanwhile, the core-shell skeleton was first suggested by Horvath & Lipsky, who showed the pellicular particles form, which were then later developed by Kirkland^3,5^. Core-shell particles, also known as superficially porous particles, consist of a solid core enveloped by a thin, porous shell. The thin shell shortens diffusion distances for analyte molecules, which typically diffuse slowly during chromatographic separation, while the presence of a non-porous core can reduce the central porous regions that are unable to participate effectively in the separation process. This improved mass transfer, combined with the larger surface area and smaller particle size of core-shell particles, enables faster separation, better peak shapes, and higher mobile phase velocities^6,7^. As interpreted by Liu et al.^7^ and according to Van Deemter plots studied by Cabooter et al.^8^ and Oláh and co-workers^9^, it can be further elucidated that core-shell configurations lead to a reduction in eddy diffusion, longitudinal diffusion, and mass transfer resistance, resulting in a decrease in the height equivalent to a theoretical plate (H) and thereby improving the overall efficiency of the column. Although significant progress has been achieved in addressing mass transfer challenges within packed particulate columns, the relentless pursuit of faster and highly efficient chromatography has necessitated the exploration of new separation media formats. Consequently, researchers have embarked on developing the monolithic silica skeleton. This silica skeleton exhibits bimodal porosity, characterized by macropores approximately 2 μm in diameter and mesopores at the nanometer scale. The large macropores facilitate unobstructed flow of the mobile phase, thereby reducing resistance. On the other hand, the mesopores contribute to a substantial surface area, ensuring high separation efficiency^10^.

Hepatitis-C virus (HCV) infection has historically been a leading cause of mortality, particularly in developing and underdeveloped nations. Although the virus was identified in the late 1990s, it is estimated to have existed for over 500 years prior to its discovery^11,12^. According to the World Health Organization (WHO), it was estimated that in 2024, the global number of chronic cases reached approximately 50 million^13^. Along the years, several medications and treatment regiments had been tried to cure HCV, however the resistant strains remained a high challenge which caused severe death rates, where about 20,000 deaths had been reported in the USA alone in 2013^14^. The real breakthrough in hepatitis C treatment came in the 2010s with the introduction of direct-acting antiviral (DAA) agents^15,16^. A recent report showed that although HCV contagion is still high with about 15.2 million cases diagnosed between 2015 and 2019, about 9.4 million cases were treated using DAA agents^17^. These novel pharmaceuticals, such as sofosbuvir, simeprevir, and ledipasvir, specifically target various stages of the HCV life cycle and have demonstrated substantially higher efficacy compared to earlier interferon-based regimens. Sofosbuvir (SFS) is a nucleotide analog inhibitor that targets the HCV NS5B polymerase, serving as a safe prodrug in combination therapy for HCV^18^. Conversely, ledipasvir (LDS) is another novel HCV NS5A inhibitor that has demonstrated efficacy against genotype 1 HCV, including activity against the S282T mutation, which is known to reduce susceptibility to SFS^19^. Despite that it has been reported that there is a markable decline in HCV mortality rates owed to the use of these DAA agents, it has been also estimated that more than 78% of HCV infections are still undiagnosed^17,20^. Thus, the need for DAA agents is still high with expected large market size^21^.

A brief literature summary of the previously reported analytical methodologies for these pharmaceutical analyses is systematically tabulated in the Supplementary Material Table S1. The review cites the reported method alongside their respective developed conditions, limits of detection (LOD) and quantification (LOQ), whenever available, and the organic solvents used in these methodologies. The compilation includes a total of 16 UV-spectrophotometric techniques, 11 high performance thin layer chromatography (HPTLC) methods, 9 voltammetry techniques (with four designated for LDS and three for SFS), proton nuclear magnetic resonance (^1^H NMR) method, 2 capillary electrophoresis techniques, and the remaining 46 methods pertain to HPLC and ultra-performance liquid chromatography (UPLC) with various detectors, including mass (MS) and ultraviolet (UV) detection. Among these reported methods, only 7 methods utilized green solvents or omitted organic solvents, of which 4 methods used electrochemical tools. Another 3 chromatographic methods were reported, of which 2 methods utilized phosphate buffers or triethylamine, which may have environmental implications. This review still underscores the importance of developing more time-efficient and environmentally sustainable methodologies.

Over the past two decades, the concept of green chemistry has acquired considerable prominence within diverse sectors of the chemical sciences. Initially, the principal aim of green chemistry was to mitigate pollution caused by industrial processes. In 1991, Anastas formally introduced this terminology as part of a research initiative undertaken by the United States Environmental Protection Agency (US-EPA). Subsequently, Anastas and Warner established twelve principles that underpin the discipline of green chemistry^22^. Analytical chemistry fulfills a dual function within the field of green chemistry. Primarily, it assesses the environmental impact and associated hazards of chemical technologies and products, thereby determining their ecological sustainability. Additionally, analytical chemistry itself is subject to the principles of green chemistry, with strategies such as reducing hazardous solvent consumption and minimizing organic waste being applicable. The advancement of innovative analytical methodologies represents a vital progression toward more environmentally sustainable practices in the chemical sciences^1^. Within the field of chromatography, two principal objectives are pursued to promote greener practices. The first objective pertains to the employment of safer mobile phase compositions, which can be linked to the 4th and 5th safety principles introduced by Anastas, related to the use of less-toxic chemical and solvents^23^. The subsequent objective seeks to reduce the consumption of organic solvents, thereby minimizing waste production (Anastas 12th principle; Prevention)^24^. For instance, ethanol is renewable and less toxic, aligning with the goals of green analytical chemistry, in comparison to the substantial quantities of acetonitrile utilized annually^25^.

The aim of this work is to compare monolithic and core–shell columns with a traditional fully porous column for the fast HPLC separation of SFS and LDS, using ethanol-water as a green mobile phase. This comparative study is expected to guide analysts in choosing between high-speed and high-resolution strategies, while demonstrating an environmentally benign method. The method’s environmental friendliness was assessed using the Analytical GREEnness (AGREE) and Modified Green Analytical Procedure Index (MoGAPI) for greenness. Also, the Blue Applicability Grade Index (BAGI) and Click Analytical Chemistry Index (CACI) were assessed for applicability and calculated for blueness. These evaluations quantitatively confirm the method’s eco-friendliness and practicality. These aims provide valuable insights for industrial quality control laboratories.

## Experimental

### Materials

**Fig. 1:**
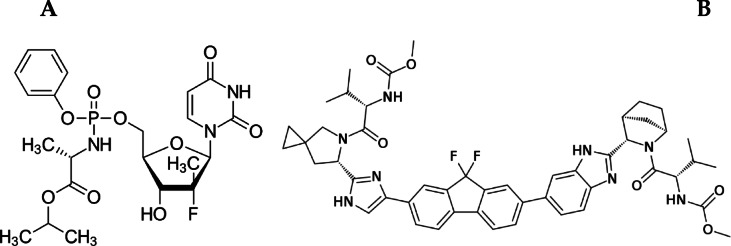
Chemical structures of SFS (**A**) and LDS (**B**).

Ethanol (EtOH; HPLC grade) was procured from Fisher Chemicals, UK. Acetic acid was of analytical grade and was obtained from Merck (Darmstadt, Germany). SFS and LDS, as acetone solvate (Chemical structures demonstrated in Fig. 1), were kindly supplied by the Egyptian International Pharmaceutical Industries Co. (EIPICO, Egypt). Harvoni^®^ tablets (400/90 mg, SFS/LDS per tablet), manufactured by Gilead Sciences (California, USA), were purchased from the local Egyptian market. Placebo tablets containing excipients (Avicel pH 102, Croscarmellose sodium, Magnesium stearate, Aerosil 200, and Opadry white) were kindly provided by EIPICO, Egypt. Purified water was freshly produced using a water purification apparatus (Millipore, Merck).

### Instruments

The HPLC used in the experiment was an Agilent 1200 series system, manufactured by Agilent Technologies (California, USA). This system comprised several components, including a solvent pump, an autosampler, a column compartment, and a UV detector (models G4220B, G4226A, G1316C and G1314E, respectively). Three stationary phase morphologies were studied. Chromolith^®^ C18 (100 × 4.6 mm) as a monolithic stationary phase was sourced from Merck, Germany. Additionally, Hypersil-BDS^®^ C18, as a totally porous particulate stationary phase (5 μm; 150 × 4.6 mm, was obtained from ThermoFisher Scientific (Massachusetts, USA). Two core-shell columns were also utilized: the Kinetex^®^ XB-C18 (5 μm; 150 × 4.6 mm) from Phenomenex (California, USA) and the Poroshell^®^ 120 EC-C18 (2.7 μm; 150 × 4.6 mm) from Agilent Technologies (California, USA).

### Standard and sample mixtures

Two different sample mixtures were investigated. The first mixture contained uracil and the two DAAs (SFS and LDS) at concentrations of 100 µg.mL⁻¹ each. This mixture was utilized to assess the performance of the columns under investigation. The second standard mixture consisted of SFS and LDS at concentrations of 4.0 mg.mL⁻¹ and 0.9 mg.mL⁻¹, respectively, serving as primary stock standard solutions for validating the quantitative method. A co-solvent mixture of ethanol: water (1:1 v/v) was used for all dilutions. All standard mixtures were stored under refrigeration at 4–8 °C. Dilutions were made from this primary stock solution to produce the working solutions.

For application in tablet dosage forms, ten tablets of Harvoni^®^ were weighed and powdered. An amount equivalent to the approximate weight of one tablet was dissolved in 10 mL of EtOH and shaken for 10 min. Subsequently, it was brought to a final volume of 100 mL with the co-solvent mixture. One mL of the solution was further diluted to 100 mL using the mobile phase.

### Chromatographic conditions

#### Procedure for stationary phase evaluation

The experiments were conducted using an isocratic mobile phase consisting of acetic acid (0.1% in water) and EtOH (6:4). The temperature of the column compartment was maintained at 30 °C, and detection was performed at a wavelength of 210 nm. Various flow rates, ranging from 0.10 mL.min^− 1^ to 1.25 mL.min^− 1^, were employed and their corresponding linear velocities were calculated. Different sample loads from the first stock mixture, ranging from 5.0 to 25.0 µL, were injected. The following parameters were determined for each column and each chromatographic run: the dead time (t_0_), representing the time taken for an unretained solute to pass from the point of injection to the detector, was determined using the unretained uracil peak. The analytes’ retention time (t_R_), the number of theoretical plates (N), and the operating backpressure (P) on each column were also measured for each chromatographic run.

#### Procedure for the quantification of SFS and LDS

Quantitation was performed on the Chromolith RP-C18 monolithic column using isocratic elution technique with a mobile phase composed of 0.1% acetic acid in water: ethanol (1:1) at a flow rate of 1.0 mL.min^− 1^. The column compartment was maintained at 30 °C, and the UV detector was set at 210 nm. The auto-sampler injection volume was 20 µL.

### Method validation

The proposed method was validated per the guidelines established by the International Council for Harmonisation (ICH)^26^. The validation process involved assessing various parameters, including linearity, accuracy, specificity, precision, reproducibility, and robustness. This evaluation aimed to determine the reliability and suitability of the method for its intended purpose.

Linearity was evaluated through the construction of a calibration curve and the determination of the coefficients of determination (R²). Each working dilution was injected 6 times, and the mean peak area was calculated and plotted against its corresponding concentration. The linearity range was established as 2.25–13.5 µg.mL^− 1^ for LDS and 10.0–60.0 µg.mL^− 1^ for SFS. Additionally, the Limits of Detection (LOD) and Quantitation (LOQ) were calculated from the calibration curve using the slope (S) and the standard deviation (ε), where LOD = 3.3ε/S and LOQ = 10ε/S.

To assess the accuracy, the placebo solution containing excipients was spiked with LDS at 5 different concentrations (4.50, 6.75, 9.00, 11.25, and 13.50 µg.mL^− 1^) and SFS at 5 different concentrations (20.00, 30.00, 40.00, 50.00, and 60.00 µg.mL^− 1^). Subsequently, precision was assessed in terms of repeatability (intra-day) and intermediate precision (inter-day) employing the same working standards. Three injections of each concentration were studied, and the average results were calculated on the same day and at three different days. The results were statistically analyzed using the relative standard deviation (RSD%). The accepted criteria for accuracy data are a ± 2% recovery from the nominal values, and precision values not exceeding ± 3% of the RSD%.

The robustness of the proposed methodology was verified through minor variations (± 2%) in the selected chromatographic conditions (flow rate, temperature, and EtOH%). Then, the impact of such changes was studied on the recovery results of a spiked placebo mixture consisting of SFS/LDS (40.00/9.00 µg.mL^− 1^, respectively).

## Results and discussion

### Stationary phase evaluation

To ensure an equitable comparison of column packings, it is imperative to select appropriate chromatographic conditions meticulously. The choice of analytes for comparison should be contingent upon the specific objectives of the analysis. In the present case, the optimal chromatographic conditions for separating the two DAAs under investigation were evaluated. Accordingly, experiments were performed using a mixture containing the same compounds of interest, SFS and LDS.

Isocratic and gradient elution are two known chromatographic techniques that differ in their solvent composition over time. Isocratic elution uses a single solvent composition throughout the separation process, which can lead to less solvent waste and lower energy consumption, enhancing its greenness and ecological impact. In contrast, gradient elution involves varying the solvent composition, which can improve separation efficiency but often requires more solvents and energy, resulting in a higher environmental footprint^27^. Therefore, while isocratic elution is generally considered more environmentally friendly due to its simplicity, reduced resource use, and time-effective where there’s no need for re-equilibration time or regulatory constraints. Moreover, Broeckhoven & Desmet^28^ discovered that the alterations in the mobile phase composition during gradient programming can affect the viscosity of the mobile phase, leading to inaccurate assessments of chromatographic column efficiency parameters like the number of theoretical plates (N) and the height equivalent to theoretical plates (H).

The performance of the stationary phase is considered based on several measurable criteria. The number of theoretical plates (N) is an important measure for chromatographic efficiency. The asymmetry factor (A_f_) indicates the peak shape and in turn can suggest issues with the chromatography process, such as column overload, and/or interaction between the analyte and stationary phase. Meanwhile, selectivity (α) and resolution (R_s_) are crucial parameters that evaluate the column’s ability to separate mixed analytes. R_s_ is a quantitative measure of the ability of a chromatographic system to separate two adjacent peaks, where α is a measure of the relative separation of two components in a mixture. Together, both can directly impact the efficiency and effectiveness in separating complex mixtures. Table 1 presents various performance characteristics obtained from the columns under examination. The optimal performance for both drugs in terms of A_f_ and N was achieved using core-shell particulate stationary phases (Kinetex^®^ and Poroshell^®^). On the Kinetex^®^ core-shell column, A_f_ and N for SFS were 0.95 and 11,360, respectively, while for LDS, these values were 1.07 and 3,095. Regarding the Poroshell^®^ core-shell, the values for SFS were 0.92 and 18,285, and for LDS, 0.99 and 3,447. Conversely, the totally porous particulate stationary phase (Hypersil-BDS^®^) exhibited high selectivity factor (α) and resolution (R_s_) values, with α at 5.0 and R_s_ at 16.23; however, it demonstrated a lower N relative to core-shell columns. This shows that the Poroshell^®^ column attained the highest resolution (17.16) for the SFS/LDS pair, despite its selectivity (α = 4.20) being lower than that of the fully porous Hypersil-BDS^®^.

**Table 1:** Performance characteristics of stationary phases under study.

Column	SFS		LDS		Selectivity (α)	Resolution (*R*_s_)
	N*	A_f_	N*	A_f_	SFS/ LDS	SFS/ LDS
Hypersil^®^	8611	0.96	3067	0.92	5.00	16.23
Kinetex^®^	11,360	0.95	3095	1.07	4.05	13.4
Poroshell^®^	18,285	0.92	3447	0.99	4.20	17.16
Chromolith^®^	6456	0.64	1905	0.99	3.22	8.15

*N calculated per 10 cm stationary phase.

The high efficiency (N) of the Poroshell^®^ column compensated for its marginally lower selectivity, as chromatographic separation is a function of both efficiency and selectivity. Meanwhile, the monolithic column displayed acceptable resolution (8.15) and selectivity (3.22), but its performance was the least favorable concerning SFS’s A_f_ (0.64) and N (6,456 and 1,905). The lower plate count for monolithic stationary phase shall be discussed in more details in Van Deemter plots (subsection Van deemter plots). Nonetheless, its performance and advantageous lower backpressure, particularly given that ethanol’s high pressure is among its disadvantages when employed as the mobile phase, render it acceptable for rapid analysis. These findings are consistent with previous studies, which indicated that core–shell and monolithic phases frequently provide enhanced efficiency or speed compared to traditional 5 μm particles^29,30^.

#### Column permeability and analysis time

According to theoretical predictions, reducing the particle size is anticipated to improve the efficiency of the packed column^31^. This improvement is indicated by an increase in the number of N and a corresponding decrease in plate height, represented by H. However, a key limitation in achieving this enhancement lies in the necessity of specialized instrumentation to counteract the escalating backpressure generated by the column as the particle size decreases. On the one hand, ethanol has a higher viscosity in comparison to acetonitrile and methanol, which further contributes to increased backpressure. On the other hand, the analysis time (t) is a critical factor influencing various facets of the analytical process. Reduced analysis time can improve the samples throughput and reduce the consumption of energy and valuable resources, taking into consideration the mobile phase flow rate and total waste produced. That in turn can improve economic and cost-effective advantages for the industry. Additionally, extended durations may potentially compromise the degradation or stability of labile analytes, thereby affecting the accuracy and reliability of the results^25,32^. In conclusion, the reduced analysis time as a strategy promotes economic efficiency and ecological greenness, paving the way for a more sustainable approach.

**Fig. 2:**
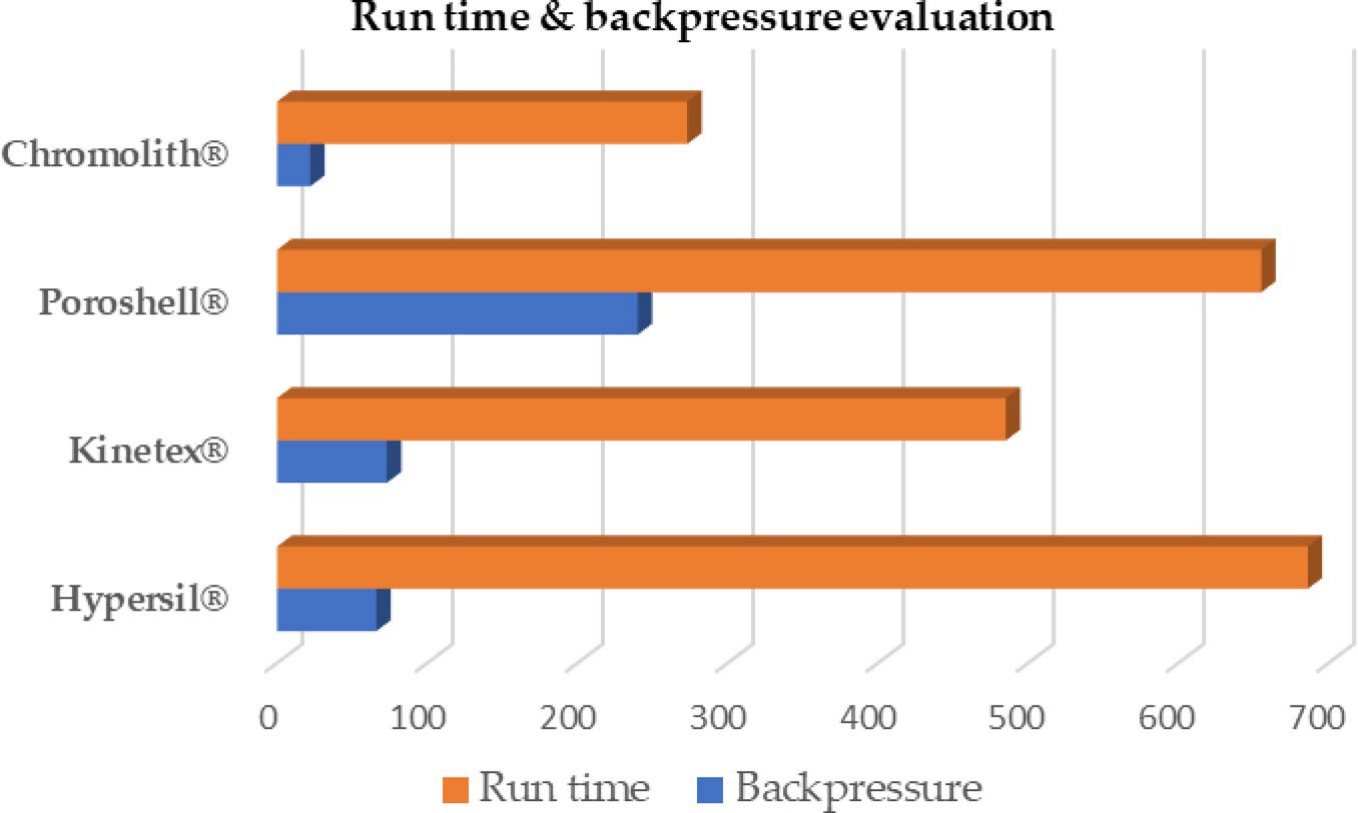
Bar chart showing the trade-off between analysis time (Seconds) and developed backpressure (Bar) on the columns under study. Mobile phase: 0.1% acetic acid in water and EtOH (6:4) at a flow rate of 1.0 mL.min^− 1^.

Figure 2 illustrates the trade-off associated with the backpressure exerted by each column (measured in bar) at a flow rate of 1 mL min^− 1^, alongside the analysis time (measured in seconds) of the last eluting peak (LDS) under identical analytical conditions. At first glance, the monolith demonstrated superior permeability, showing lower pressure at approximately three minutes of runtime. In contrast, the particulate columns, especially Poroshell^®^ and Hypersil^®^, experienced higher backpressures. The results could be attributed to the monolithic macroporous structure, which facilitates a more rapid mobile phase flow compared to particulate stationary phase morphologies, together with its shorter dimension. Moreover, the highest resistance was observed in the Poroshell^®^ column, which is consistent with its smallest particle size. Meanwhile, when comparing the same particle size among fully porous Hypersil^®^ and core-shell Kinetix^®^ columns, it was unexpectedly observed that fully porous particles demonstrate higher permeability than the core-shell counterparts. This observed discrepancy can be explained, according to Destefano^33^, as the variation in particle size distribution (PSD) between the two types of particulate columns may have exerted an influence. Core-shell particles exhibit a narrower range of particle size distribution compared to fully porous particles as a fundamental result of the manufacturing process, resulting in a more uniform homogenous packing^34,35^. This in turn reduces the irregularity of the flow paths, increases flow resistance, and hence leads to higher backpressure^36^. Ultimately, the monolithic column’s continuous macroporous structure provides more uniform distribution & easy flow, resulting in significantly lower backpressure. This in turn allows for faster flow rates and hence shorter analysis times without exceeding system pressure limits. Meanwhile, the fully porous 5 μm column, although having lower backpressure than the core-shell, yielded the longest analysis times due to its longer diffusion paths and hence longer mass transfer as shall be discussed in Van Deemter plots next subsection.

#### Van deemter plots

**Fig. 3:**
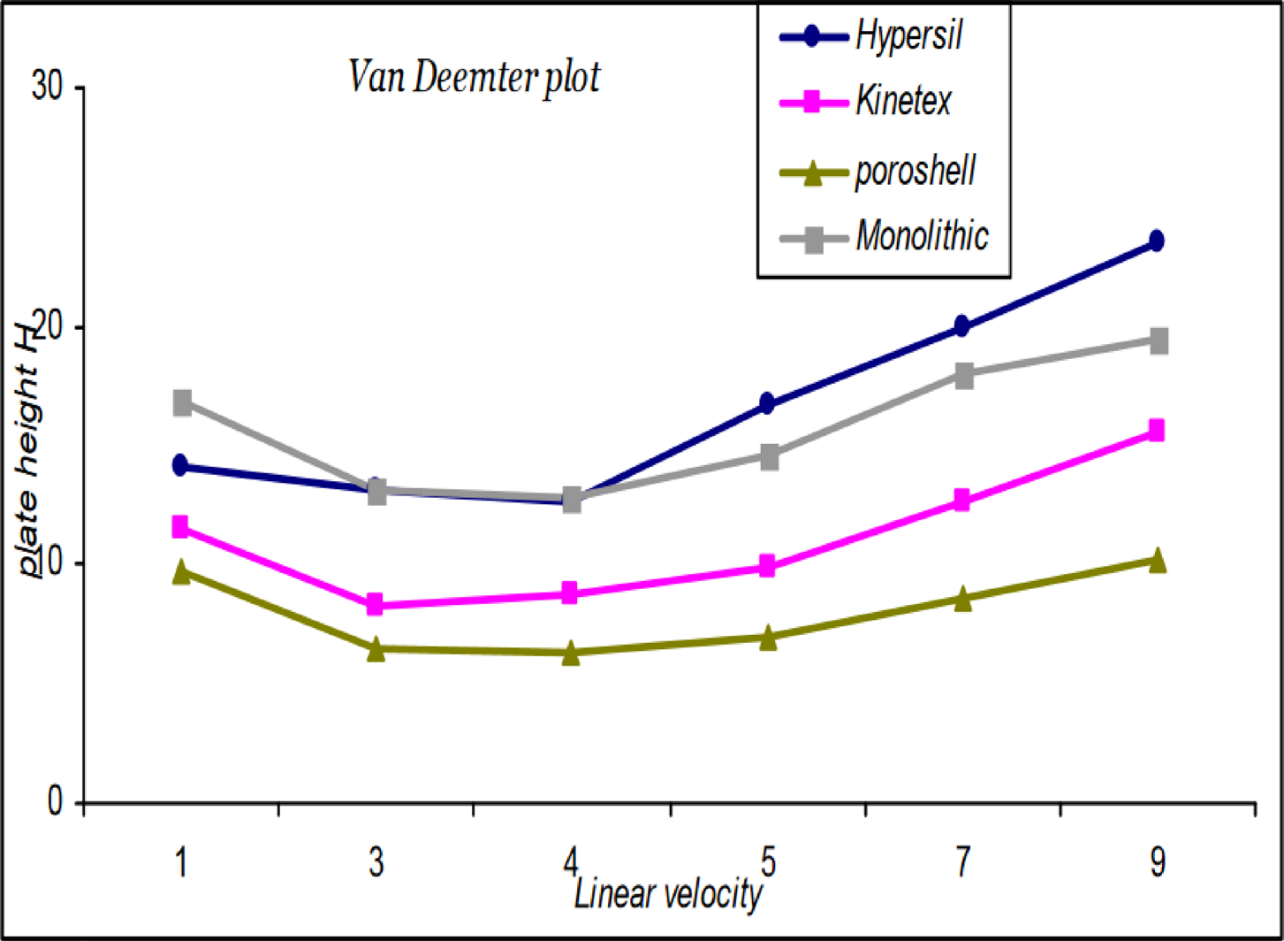
Van Deemter plot for SFS peak on different stationary phase morphologies under study.

Van Deemter plots were generated for the stationary phases studied, using isocratic elution to evaluate their performance. The linear velocity (cm/min) and the height equivalent to theoretical plates (H; µm) for the SFS peaks were determined. Figure 3 presents the comparison of the Van Deemter plots for the studied columns. The findings demonstrate that core-shell particulate columns possess the lowest plate height, H, at the optimal velocity among the stationary phases examined, thereby affirming their superior efficiency. This advantage is attributable to both reduced eddy diffusion (A term) resulting from the narrower particle size distribution and, notably, to minimized mass transfer resistance (C term) attributable to their distinctive core-shell structure. The monolithic and fully porous columns display similar H values at their respective optimal velocities, as their open pore and particle architecture exert minimal influence on longitudinal diffusion (B term) at these flow rates. Meanwhile, totally porous particles have longer diffusion paths, where solutes must diffuse into the particles to access the porous stationary phase inside. This in turn leads to very slow mass transfer and high C-term, as expressed in terms of total runtime (Fig. 2) compared to the same particle size core-shell particles (Kinetex^®^ column), which have shorter diffusion paths thanks to their solid core. It’s worth mentioning that (as shown in Fig. 3) at higher flow rates, where the C-term dominates, molecules in the mobile phase that are flowing through the macropores move ahead of the molecules that are still slowly diffusing within the skeletons. This causes band broadening, which directly translates to a higher H and a lower number of theoretical plates compared to a column packed with small, modern particles. Thus, at elevated velocities, the monolith’s extensive through-pores facilitate rapid flow of the mobile phase, can advantageously constrain the increase in H attributable to the C term (as shown relative to the totally porous particles Hypersil^®^ column). This observation elucidates why monolithic columns can be operated at higher flow rates without significant compromise to efficiency, whereas fully porous columns experience more pronounced efficiency losses due to mass transfer limitations. Thus, if one’s priority is achieving the highest efficiency per unit time, the core-shell column is preferable, whereas the monolithic column provides efficiency comparable to that of a traditional fully porous column, but with the benefits of higher permissible flow and the potential for faster HPLC analyses^6,7,37^.

### Method development

Given that SFS and LDS molecules are chromophores, solutions containing each drug at a concentration of 0.1 mg mL^− 1^ were subjected to ultraviolet (UV) spectroscopic analysis. The SFS exhibited maximum absorbance at 261 ± 1 nm, while the LDS showed a maximum absorbance at 334 ± 1 nm. Considering that their respective λ_max_ values are sufficiently distant to preclude the use of a single wavelength, a wavelength of 210 nm was selected to enable the simultaneous detection of both pharmaceuticals with high sensitivity. At 210 nm (far-UV), both analytes demonstrate adequate absorption, and the ethanol/water solvent system exhibits minimal background interference, given that this wavelength surpasses their UV cut-off thresholds^38^.

Water, as a pure aqueous mobile phase part, didn’t provide good reproducibility in the retention times of the analytes. Therefore, it was prepared as a 0.1% acetic acid solution, which improved reproducibility and peak shape and enhanced the resolution between the two analytes. The use of acetic acid also offers a greener alternative compared to phosphate buffer and triethylamine approaches previously mentioned in the introduction. Moreover, the chromatographic performance study of different stationary phase morphologies showed that the monolithic skeleton can accommodate higher pressure, and higher flow rates without considerable loss of chromatographic performance as indicated by H and N. Thanks to that, the monolithic skeleton was chosen as a stationary phase, and since it could accommodate more viscous mobile phase combinations without causing higher backpressure or considerable efficiency loss, a 10% increase of the green organic modifier, ethanol, was used. This increase shortened the analysis time causing faster elution of the analytes under study in 3 min runtime while keeping the chromatographic separation efficiency.

**Fig. 4:**
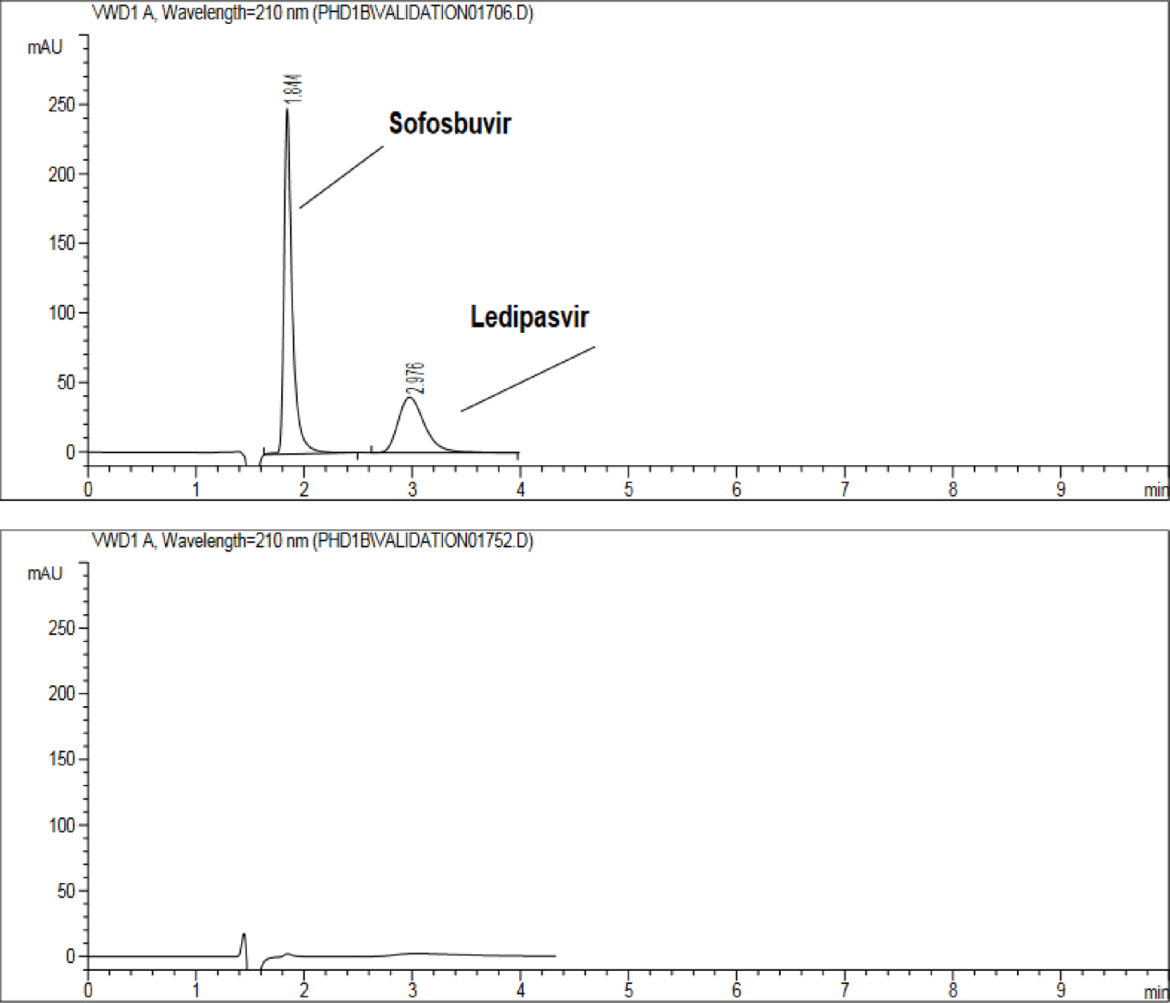
Chromatogram showing separation of SFS and LDS (**A**) and placebo (**B**) injections. Mobile phase: 0.1% acetic acid in water and EtOH (1:1) at flow rate 1.0 mL.min^− 1^.

Figure 4 depicts a representative chromatogram acquired under these optimized conditions using the monolithic column. It features well-resolved peaks (α and R_s_ equal to 3.5 and 4.0) for SFS (1.84 min) and LDS (2.98 min), highlighting rapid analysis without compromising efficiency.

### Method validation

#### System suitability parameters, selectivity and regression

The system suitability parameters for the proposed chromatographic conditions were calculated for the two analytes injected under the defined isocratic conditions. Subsequently, selectivity (α), retention time (t_R_), resolution (R_s_), and theoretical plates (N) were determined. The N values of SFS and LDS were 3,600 and 760, respectively, with a selectivity of 3.5 and a resolution of 4.0. The regression data indicate excellent linearity (R2 > 0.99) within the specified range. LOD was 0.23 µg.mL^− 1^ for LDS and 0.72 µg.mL^− 1^ for SFS, while LOQ was 0.72 µg.mL^− 1^ (LDS) and 2.18 µg.mL^− 1^ (SFS), confirming the method’s sensitivity to minute concentrations of SFS and LDS. These results are presented in Table 2. Regarding selectivity, an injection solution containing placebo tablets prepared identically to the tablet samples was examined, revealing no significant interference from excipients at the retention times of LDS or SFS (Fig. 4).

**Table 2:** Linearity, calibration data, and system suitability for SFS and LDS determination using the proposed HPLC method (*n* = 6).

Parameters	SFS	LDS
Linearity range (µg.mL^-1^)	10.00–60.00	2.25–13.50
Regression equation	Y = 30.8X + 86.9	Y = 66.6X + 62.8
Coefficient of determination (R^2^)	0.999	0.999
LOD (µg.mL^-1^)	0.72	0.23
LOQ (µg.mL^-1^)	2.18	0.72
t_R_ ± SD (min.)	1.85 ± 0.1	2.97 ± 0.3
N	7600	2760
α	–	3.5
R_s_	–	4.0

#### Accuracy and precision

The accuracy (Table 3) and inter- and intra-day assay precision results, determined by analyzing QC samples at low, medium, and high concentration levels on the same day and different days (Table 4) were examined. The overall accuracy results expressed in (Recovery% ± RSD%) are 99.4 ± 2.1 and 100.9 ± 2.2 for SFS and LDS, respectively.

**Table 3:** Accuracy results for SFS and LDS determination (*n* = 3).

Analyte	Concentration used (µg.mL^-1^)	Accuracy (Recovery% ±RSD)
	20	96.9 ± 0.24%
SFS	30	102.5 ± 0.64%
	40	100.3 ± 0.16%
	50	98.3 ± 0.15%
	60	98.9 ± 0.62%
	4.5	98.0 ± 5.0%
LDS	6.75	101.2 ± 1.7%
	9.0	103.8 ± 0.77%
	11.25	99.6 ± 0.42%
	13.5	102.1 ± 0.12%

**Table 4:** Inter- and Intra-day precision results (*n* = 3).

Concentration used (µg.mL^-1^)	Inter-day (Recovery% ±RSD%)	Intra-day (Recovery% ±RSD%)
SFS		
30	102.3 ± 2.3%	102.8 ± 0.8%
40	101.3 ± 1.1%	100.3 ± 0.1%
50	99.6 ± 1.0%	98.4 ± 0.2%
LDS		
6.75	96.9 ± 4.2%	101.5 ± 1.1%
9.0	101.0 ± 2.4%	103.5 ± 0.5%
11.25	97.3 ± 1.6%	99.4 ± 0.2%

The stability of the sample solutions was determined by monitoring the areas of peaks corresponding to SFS and LDS over 5 days at room temperature (15–25 °C) and for 7 days in the refrigerator (4–8 °C).

#### Robustness

The minor variations in flow rate, temperature, and ethanol percentage demonstrated that the recovery% was not affected by more than 5%, indicating that the method is robust and suitable for routine analysis.

### Method’s application

The proposed method was successfully applied to the determination of SFS and LDS in Harvoni^®^ tablets. The results were compared statistically to those obtained using a reference method by Hemdan and Eissa^39^ via Student’s t-test (Table 5). The results indicated that there were no notable differences between the developed HPLC method and the reported methodology.

**Table 5:**
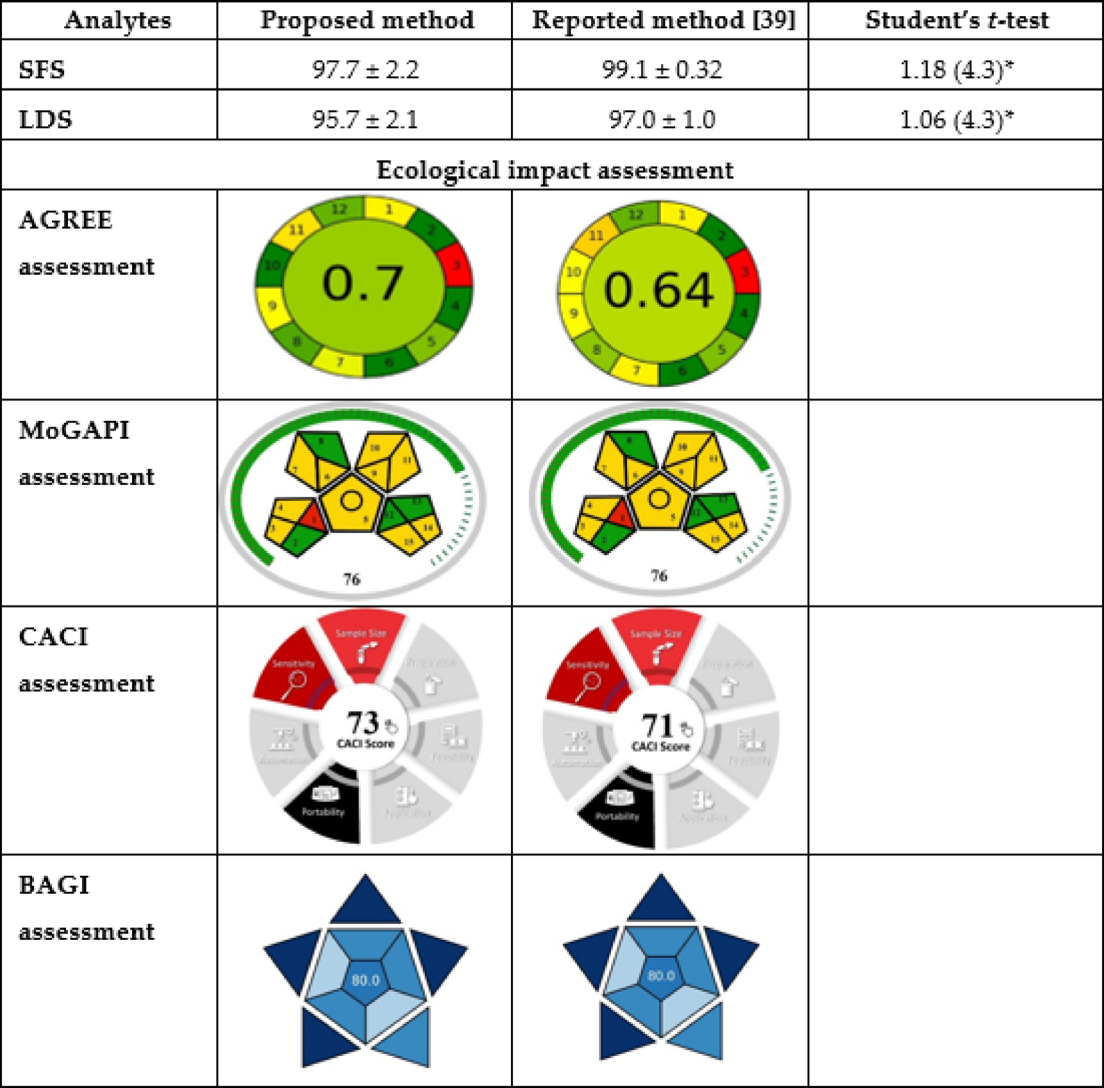
Comparison between the results for determination of SFS and LDS using the proposed HPLC and reported spectroscopic methods together with greenness assessment.

*Theoretical values of the student t-test at (P=0.05) where (n = 3).

### Blueness and greenness assessment

A new generation of metric-based tools has emerged in analytical chemistry, providing comprehensive, multidimensional assessments of methodologies that extend far beyond traditional performance criteria. The Analytical GREEnness (AGREE) metric^40^ exemplifies this progress by evaluating the environmental sustainability of analytical methods according to all twelve principles of green analytical chemistry. Its standardized 0–1 scoring system and intuitive pictogram allow for quick, holistic evaluation of both overall greenness and specific methodological strengths and weaknesses. Building on visual frameworks like GAPI, the Modified Green Analytical Procedure Index (MoGAPI)^41^ advances environmental assessment by introducing an overall quantitative score, facilitating easier comparison and classification of methods based on their total greenness, while maintaining the visual clarity and user-friendliness of GAPI^42^. Recognizing that environmental impact is only one dimension of method value, other tools have emerged to address additional practical needs. The Blue Applicability Grade Index (BAGI)^43^ systematically evaluates real-world applicability by focusing on operational efficiency, productivity, and user-friendliness through transparent scoring of ten key attributes and a clear pictogram. The Click Analytical Chemistry Index (CACI)^44^ further extends this perspective by explicitly prioritizing operational usability, rapid analysis, and adaptability, drawing inspiration from the core principles of click chemistry: simplicity, efficiency, and reliability. CACI incorporates a range of practical criteria, including sample size, preparation demands, cost, automation, and speed, enabling researchers to optimize and compare methods for both laboratory and field use. Collectively, these advanced metrics support the development and selection of analytical methodologies that are not only environmentally sustainable but also practically accessible and high-performing across a broad spectrum of analytical challenges.

The proposed method was evaluated using these four metrics and was compared to the most similar method among those reported, which was by Hemdan and Eissa^39^.

Both methods achieved a score of 76 on MoGAPI and 80 on BAGI, while the proposed method obtained 0.7 on AGREE, with the reported score being 0.64. On CACI, the proposed method scored 73, whereas the reported score was 71. The scores are presented in (Table 5). These scores consider both methods practical, with BAGI scores exceeding 60 and CACI scores surpassing 50, and environmentally friendly, as denoted by the green metrics pictograms.

The penalties associated with the reported method in MoGAPI were due to being offline (segment 1), necessitating transport and storage (segments 3 and 4), simple sample preparation (segment 5), microextraction (segment 6), utilization of green solvents (segment 7), considerations regarding ethanol safety and quantities (9–11), and waste treatment (segment 15). The proposed method incurred identical penalties, resulting in an equivalent score; however, the AGREE metric, which delineates both shared and distinct aspects of green analytical chemistry principles, elucidates subtle differences. In the AGREE metric, both shared the same penalties in segments 1 and 3 (offline analysis), considering the HPLC autosampler as semi-automatic (segment 5), with the amount of waste being ≤ 5 mL (segment 7), the energy of the HPLC (segment 9), and the highly flammable nature of ethanol (segment 12). The distinctions between the two are observed in segments 8, 10, and 11. The variation in segment 8 is attributable to differing throughputs; the reported method can analyze 12 samples per hour, whereas the proposed method can analyze 20 samples per hour. The difference in segment 10 stems from the use of phosphate buffer in the reported method, which results in not all reagents being biobased, in contrast to the proposed method. Lastly, in segment 11, the volume of ethanol used per run (calculated as % in mobile phase × flow rate × run time) was 2 mL in the reported method, compared to 1.5 mL in the proposed method. The accumulation of these differences contributed to the variation in the scores, favoring the proposed.

Regarding practicality, both methods incurred identical penalties in BAGI (segments 1–5 and segment 9) due to their quantitative nature (both utilizing UV detectors rather than PDA), involving two analytes from the same class, simple instrumentation and sample preparation, with HPLC being regarded as semi-automatic. The differences observed in CACI primarily stem from variations in sample preparation and analysis durations. BAGI considers the throughput of the methodology exclusively; since both methods exceeded 10 samples per hour, they did not incur penalties. Conversely, CACI includes a broader spectrum of scoring penalties that account for a wide range of time consumption. The reported method sample preparation time is approximately 35 min (comprising 30 min of sonication and 5 min of weighing and filtering, as per criterion number 2 in CACI), and 40 min under criterion number 8 of CACI (including 35 min for sample preparation and 5 min for the run time). The proposed method, however, requires roughly 15 min (10 min of shaking and 5 min of weighing and filtering) for sample preparation, along with 18 min of sample analysis time (including 15 min for sample preparation and 3 min for run time). This short comparison encompasses the use of different metrics to cover the entire spectrum of analytical principles being assessed, as different metrics share some aspects while also addressing principles from different perspectives, thereby providing a clearer overview of the methods’ performance.

## Conclusions

This research was conducted to facilitate the practical implementation of performance comparison, assisting in the selection of the most appropriate stationary phase that provides an optimal balance between the analysis’s objective, reduced analysis time, and separation efficiency. The study highlights the impact of column morphology on key performance metrics, noting that core-shell particles optimize efficiency and sensitivity. On the other hand, the monolithic column, although possessing a lower number of theoretical plates, demonstrated adequate selectivity and superior permeability, thereby facilitating the use of environmentally friendly solvents such as ethanol. This nuanced understanding can serve to guide future method development for multi-analyte separations. The fast, isocratic, three-minute analysis of Sofosbuvir and Ledipasvir, with high accuracy and robustness, enables exceptionally high throughput while minimizing operational costs, rendering it particularly advantageous for industrial quality control settings. Furthermore, the use of standard HPLC equipment without reliance on expensive UPLC systems and the substitution of acetonitrile or methanol with ethanol as the mobile phase confer considerable advantages, promoting both economic efficiency and environmental sustainability. Collectively, these measures enhance laboratory safety, practicality, and compliance with the green and blue analytical chemistry principles, as demonstrated by the different good scores obtained.

## Supplementary Information

Below is the link to the electronic supplementary material.

Supplementary Material 1

## Data Availability

All data is available from the corresponding author upon reasonable request.

## References

[1] Ibrahim, A. E., Taher, M. F., Gindy, E., Ibrahim, E. A. & A. & Novel green HPTLC and organic solvent-free micellar LC methods for the simultaneous determination of ozenoxacin and benzoic acid; greenness assessment and applications. *Sustainable Chem. Pharm.* **36**, 101277. 10.1016/j.scp.2023.101277 (2023).

[2] Gumułka, P., Żandarek, J., Dąbrowska, M. & Starek, M. UPLC technique in pharmacy—an important tool of the modern analyst. *Processes* **10**, 2498 (2022).

[3] Jaag, S., Wen, C., Peters, B. & Lämmerhofer, M. Kinetic performance comparison of superficially porous, fully porous and monolithic reversed-phase columns by gradient kinetic plots for the separation of protein biopharmaceuticals. *J. Chromatogr. A*. **1676**, 463251 (2022). 35752149 10.1016/j.chroma.2022.463251

[4] Minakuchi, H., Nakanishi, K., Soga, N., Ishizuka, N. & Tanaka, N. Octadecylsilylated porous silica rods as separation media for reversed-phase liquid chromatography. *Anal. Chem.* **68**, 3498–3501 (1996). 21619283 10.1021/ac960281m

[5] Horvath, C. & Lipsky, S. Column design in high pressure liquid chromatography. *J. Chromatogr. Sci.* **7**, 109–116 (1969).

[6] Liekens, A., Denayer, J. & Desmet, G. Experimental investigation of the difference in B-term dominated band broadening between fully porous and porous-shell particles for liquid chromatography using the effective medium theory. *J. Chromatogr. A*. **1218**, 4406–4416 (2011). 21628063 10.1016/j.chroma.2011.05.018

[7] Liu, Z. et al. Preparation of silica-based superficially porous silica and its application in enantiomer separations: a review. *J. Anal. Test.* **5**, 242–257 (2021).

[8] Cabooter, D. et al. Detailed characterization of the kinetic performance of first and second generation silica monolithic columns for reversed-phase chromatography separations. *J. Chromatogr. A*. **1325**, 72–82. 10.1016/j.chroma.2013.11.047 (2014). 24342534 10.1016/j.chroma.2013.11.047

[9] Oláh, E., Fekete, S., Fekete, J. & Ganzler, K. Comparative study of new shell-type, sub-2 µm fully porous and monolith stationary phases, focusing on mass-transfer resistance. *J. Chromatogr. A*. **1217**, 3642–3653 (2010). 20409553 10.1016/j.chroma.2010.03.052

[10] Hefnawy, M. et al. Trends in monoliths: Packings, stationary phases and nanoparticles. *J. Chromatogr. A*, 463819 (2023). 10.1016/j.chroma.2023.463819 36724721

[11] Roudot-Thoraval, F. Epidemiology of hepatitis C virus infection. *Clin. Res. Hepatol. Gastroenterol.* **45**, 101596. 10.1016/j.clinre.2020.101596 (2021). 33610022 10.1016/j.clinre.2020.101596

[12] El-Derany, M. O. Chemerin is an indispensable pre-treatment predictor of sofosbuvir, pegylated interferon-alpha and ribavirin outcomes in chronic hepatitis c Egyptian patients. *Al-Azhar J. Pharm. Sci.* **60**, 111–121 (2019).

[13] WHO. World Health Organization, (2024).

[14] Ibrahim, A. E., Hashem, H., Elhenawee, M. & Saleh, H. Comparison between core–shell and totally porous particle stationary phases for fast and green LC determination of five hepatitis-C antiviral drugs. *J. Sep. Sci.* **41**, 1734–1742 (2018). 29297968 10.1002/jssc.201701263

[15] Wahsh, E. Real life Egyptian experience of combination therapy (simeprevir/sofosbuvir) in experienced Non cirrhotic Hcv patients. *Al-Azhar J. Pharm. Sci.* **55**, 128–135 (2017).

[16] Abdel-Lateef, M. A., Omar, M. A., Ali, R. & Derayea, S. M. Micellar spectrofluorimetric protocol for the innovative determination of HCV antiviral (daclatasvir) with enhanced sensitivity: application to human plasma and stability study. *Spectrochim. Acta Part A Mol. Biomol. Spectrosc.* **206**, 57–64 (2019). 10.1016/j.saa.2018.07.101 30081268

[17] Cui, F. et al. Global reporting of progress towards elimination of hepatitis B and hepatitis C. *Lancet Gastroenterol. Hepatol.* **8**, 332–342 (2023). 36764320 10.1016/S2468-1253(22)00386-7

[18] El-Gebaly, A. A. & Predictive Comparative study via viral and biochemical measurements for responders and Non-Responders Egyptian hepatitis C patients to Daclatasvir plus Sofosbuvir therapy. *Al-Azhar J. Pharm. Sci.* **58**, 37–59 (2018).

[19] Brown, D. G. & Wobst, H. J. A decade of FDA-approved drugs (2010–2019): trends and future directions. *J. Med. Chem.* **64**, 2312–2338 (2021). 33617254 10.1021/acs.jmedchem.0c01516

[20] Abdel-Lateef, M. A., Omar, M. A., Ali, R. & Derayea, S. M. Xanthene based spectroscopic probe for the analysis of HCV antiviral, Daclatasvir dihydrochloride, through feasible complexation reaction. *Microchem. J.* **145**, 672–675 (2019).

[21] Barber, M. J., Gotham, D., Khwairakpam, G. & Hill, A. Price of a hepatitis C cure: cost of production and current prices for direct-acting antivirals in 50 countries. *J. Virus Eradic.*. **6**, 100001. 10.1016/j.jve.2020.06.001 (2020). 10.1016/j.jve.2020.06.001 PMC7646676 33251019

[22] Anastas, P. T. & Warner, J. C. *Green Chemistry: Theory and Practice* (Oxford University Press, 2000).

[23] Anastas, P. & Eghbali, N. Green chemistry: principles and practice. *Chem. Soc. Rev.* **39**, 301–312. 10.1039/B918763B (2010). 20023854 10.1039/b918763b

[24] El Deeb, S., Abdelsamad, K. & Parr, M. K. Greener and whiter analytical chemistry using cyrene as a more sustainable and Eco-Friendlier mobile phase constituent in chromatography. *Pharmaceuticals* **16**, 1488 (2023). 37895959 10.3390/ph16101488 PMC10609853

[25] Alamir, S. G., Magdy, N., Hussein, L. A., Ibrahim, A. E. & Fares, N. V. Experimental design utilizing mixed micellar monolithic liquid chromatography and ethanol for rapid simultaneous determination of four antipsoriatics: A Blue–Green approach. *Sep. Sci. PLUS*. **8**, e70003. 10.1002/sscp.70003 (2025).

[26] Guideline. in International Conference on Harmonization, Geneva, Switzerland 11–12. (2005).

[27] Schellinger, A. P. & Carr, P. W. Isocratic and gradient elution chromatography: A comparison in terms of speed, retention reproducibility and quantitation. *J. Chromatogr. A*. **1109**, 253–266. 10.1016/j.chroma.2006.01.047 (2006). 16460742 10.1016/j.chroma.2006.01.047

[28] Broeckhoven, K. & Desmet, G. Methods to determine the kinetic performance limit of contemporary chromatographic techniques. *J. Sep. Sci.* **44**, 323–339 (2021). 32902146 10.1002/jssc.202000779

[29] Ahmed, A., Skinley, K., Herodotou, S. & Zhang, H. Core–shell microspheres with porous nanostructured shells for liquid chromatography. *J. Sep. Sci.* **41**, 99–124. 10.1002/jssc.201700850 (2018). 28994266 10.1002/jssc.201700850

[30] Ehab Ibrahim, A., Hashem, H., Elhenawee, M. & Saleh, H. Monolithic and core-shell particles stationary phase morphologies in protein analysis; peptide mapping of erythropoietin hormone and determination of Carbetocin. *Ann. Pharm. Fr.* **78**, 206–216. 10.1016/j.pharma.2020.01.008 (2020). 32247515 10.1016/j.pharma.2020.01.008

[31] Žuvela, P. et al. Column characterization and selection systems in reversed-phase high-performance liquid chromatography. *Chem. Rev.* **119**, 3674–3729 (2019). 30604951 10.1021/acs.chemrev.8b00246

[32] Ibrahim, A. E., Maged, K., Elhenawee, M. & El-Hay, S. S. A. Integrating micellar HPLC and green analytical chemistry tools in greenness assessment of five commonly Co-formulated antidiabetic drugs. *Sustainable Chem. Pharm.* **35**, 101199. 10.1016/j.scp.2023.101199 (2023).

[33] DeStefano, J. J., Schuster, S. A., Lawhorn, J. M. & Kirkland, J. J. Performance characteristics of new superficially porous particles. *J. Chromatogr. A*. **1258**, 76–83 (2012). 22939204 10.1016/j.chroma.2012.08.036 PMC3606024

[34] Gritti, F. & Guiochon, G. The mass transfer kinetics in columns packed with Halo-ES shell particles. *J. Chromatogr. A*. **1218**, 907–921 (2011). 21236440 10.1016/j.chroma.2010.12.046

[35] Kirkland, J. J., Langlois, T. J. & DeStefano, J. J. Fused core particles for HPLC columns. *Am. Lab.* **39**, 18–21 (2007).

[36] Gritti, F. et al. Performance of columns packed with the new shell particles, Kinetex-C18. *J. Chromatogr. A*. **1217**, 1589–1603 (2010). 20116065 10.1016/j.chroma.2009.12.079

[37] Cabooter, D. et al. Measurement and modelling of the intra-particle diffusion and b-term in reversed-phase liquid chromatography. *J. Chromatogr. A*. **1637**, 461852 (2021). 33412290 10.1016/j.chroma.2020.461852

[38] Ruwoldt, J., Tanase-Opedal, M. & Syverud, K. Ultraviolet spectrophotometry of lignin revisited: exploring solvents with low Harmfulness, lignin Purity, Hansen solubility Parameter, and determination of phenolic hydroxyl groups. *ACS Omega*. **7**, 46371–46383. 10.1021/acsomega.2c04982 (2022). 36570215 10.1021/acsomega.2c04982 PMC9773929

[39] Hemdan, A. & Eissa, M. S. Simultaneous chromatographic analysis of Sofosbuvir/Ledipasvir in their combined dosage form: an application to green analytical chemistry. *J. Anal. Sci. Technol.* **10**, 39. 10.1186/s40543-019-0197-x (2019).

[40] Pena-Pereira, F., Wojnowski, W. & Tobiszewski, M. AGREE—Analytical greenness metric approach and software. *Anal. Chem.* **92**, 10076–10082 (2020). 32538619 10.1021/acs.analchem.0c01887 PMC7588019

[41] Mansour, F. R., Płotka-Wasylka, J., Locatelli, M. & Modified, G. A. P. I. (eds) (MoGAPI) Tool and Software for the Assessment of Method Greenness: Case Studies and Applications. *Analytica* 5, 451–457 (2024).

[42] Ayad, M. M., Hosny, M. M., Ibrahim, A. E., El-Abassy, O. M. & Belal, F. F. An eco-friendly micellar HPLC method for the simultaneous determination of triamterene and xipamide in active pharmaceutical ingredients and marketed tablet dosage form. *Acta Chromatographica*. **33**, 51–56 (2021).

[43] Manousi, N., Wojnowski, W., Płotka-Wasylka, J. & Samanidou, V. Blue applicability grade index (BAGI) and software: a new tool for the evaluation of method practicality. *Green Chem.* **25**, 7598–7604. 10.1039/D3GC02347H (2023).

[44] Mansour, F. R., Bedair, A. & Locatelli, M. Click analytical chemistry index as a novel concept and framework, supported with open source software to assess analytical methods. *Adv. Sample Prepar.*. **14**, 100164. 10.1016/j.sampre.2025.100164 (2025).

