# Supplementary material for: Exploring stationary phase morphologies as pathways to greener and faster LC for analyzing sofosbuvir and ledipasvir
Source: Sci Rep. 2025 Dec 15;16:1847. doi: 10.1038/s41598-025-31605-x (PMC12804721; doi:10.1038/s41598-025-31605-x)
Supplement: Supplementary file 1 — Supplementary Material 1 [file 41598_2025_31605_MOESM1_ESM.pdf]

Supplementary Materials

Exploring Stationary Phase Morphologies as Pathways to Greener and Faster LC for Analyzing Sofosbuvir and Ledipasvir

Adel Ehab Ibrahim[1,2*], Samy G. Alamir[1,3], Baher I Salman[4], Sami El Deeb[5*], and Ahmed Al-Harrasi[1*]

[1] Natural and Medical Sciences Research Center, University of Nizwa, P.O. Box 33, Birkat Al Mauz, Nizwa 616, Sultanate of Oman; adel@unizwa.edu.om (A.I); aharrasi@unizwa.edu.om (A.H)

[2] Pharmaceutical analytical chemistry department, Faculty of Pharmacy, Port-Said University, Port-Said 42511, Egypt

[3] Pharmaceutical Analytical Chemistry Department, Faculty of Pharmacy, Ain Shams University, Abassia 11566, Cairo, Egypt; sami.goerge@pharma.asu.edu.eg (S.A)

[4] Pharmaceutical Analytical Chemistry Department, Faculty of Pharmacy, Al-Azhar University, Assiut branch, Assiut 71524, Egypt; bahersalman@azhar.edu.eg (B.S)

[5] Institute of Medicinal and Pharmaceutical Chemistry, Technische Universitaet Braunschweig, 38106 Braunschweig, Germany; s.eldeeb@tu-bs.de

***Corresponding author:** adel@unizwa.edu.om; s.eldeeb@tu-bs.de; aharrasi@unizwa.edu.om

Supplementary materials Table S1: Literature review for the analytical methods for determination of SFS/LDS combination

Reported method	Technique	Application	Organic Solvents	Conditions	Linear range	LOD	LOQ
[1]	UV spectrophotometric	Tablet Dosage Form	Methanol	UV direct method at 333 nm (LPV) & wavelength - intersection ratio method (SBV)	3 - 18 µg/mL (LPV)<br>11 - 110 µg/mL (SBV)	3 µg/mL (SBV)<br>0.5 µg/mL (LPV)	11 µg/mL (SBV)<br>3 µg/mL (LPV)
[2]	RP - HPLC	Tablet Dosage Form & Dissolution	Acetonitrile	C8 column, mobile phase ammonium acetate buffer: ACN (35:65 %). Flow rate of 0.7mL/min. UV detector at 245 nm.	8 - 96 µg/mL (SBV)<br>1.8 - 21.6 µg/mL (LPV)	0.485 µg/mL (SBV)<br>0.175 µg/mL (LPV)	1.619 µg/mL (SBV)<br>0.586 µg/mL (LPV)
[3]	LC-MS/MS	Human plasma	Methanol<br>Acetonitrile<br>Diethyl ether<br>Dichloromethane	C18 column, mobile phase ammonium acetate: ACN:0.1% methanolic formic acid (12:25:63). Flow rate 0.6ml/min. 40°C	0.5 - 2500 ng/mL (SBV)<br>5 - 2100 ng/mL (LPV)	- - -	0.5 ng/mL (SBV)<br>5 ng/mL (LPV)
[4]	RP - HPLC	Tablet Dosage Form & Dissolution	Methanol<br>Acetonitrile<br>Hexane sulfonate	C18 column, mobile phase phosphate buffer, and hexane sulfonate: ACN (50:50). Flow rate 1.5ml/min. UV detector at 254 nm.	40 - 500 µg/mL (SBV)<br>30 - 300 µg/ mL (LPV)	4.14 µg/mL (SBV)<br>3.64 µg/ mL (LPV)	12.54 µg/mL (SBV)<br>11.03 µg/ mL (LPV)
[5]	HPTLC - densitometric Scanner	Tablet Dosage Form	Methylene chloride<br>Methanol<br>Ethyl acetate	Silica gel 60 F254, mobile phase methylene chloride: methanol: ethyl acetate: ammonia (25%) (6:1:4:1).	100 - 3000 ng/spot (SBV)<br>50 - 3000 ng/spot (LPV)	22.5 ng/spot (SBV)<br>15.80 ng/spot (LPV)	67.50 ng/spot (SBV)<br>47.50 ng/spot (LPV)
[6]	UV spectrophotometric	Tablet Dosage Form	Methanol	UV direct method at 333 nm (LPV)<br>For (SBV):<br>3rd derivative<br>Derivative ratio<br>Ratio difference method<br>Ratio subtraction method	5 - 35 µg/mL (SBV)<br>4 - 14 µg/mL (LPV)	0.028 µg/mL (SBV)<br>0.020 µg/mL (LPV)	0.085 µg/mL (SBV)<br>0.062 µg/mL (LPV)
[7]	UV spectrophotometric (Chemometric)	Tablet Dosage Form	Acetonitrile	Partial least squares (PLS), different families of continuous wavelet transform (CWT), and first derivative spectrophotometry (DS) techniques.	24 - 64 µg/mL (SBV)<br>6 - 16 µg/mL (LDV)	0.251 µg/mL (SBV)<br>0.106 µg/mL (LPV)	0.837 µg/mL (SBV)<br>0.352 µg/mL (LPV)
[8]	RP - HPLC	Dosage form & degradation	Acetonitrile	C18 column, mobile phase ACN & TEA (50:50), flow rate 1ml/min. UV 227nm. 30°C	1 - 15 µg/ml (SBV)<br>0.25 - 3.75 µg/ml (LPV)	0.25 µg/ml (SBV)<br>0.0625 µg/ml (LPV)	0.5 µg/ml (SBV)<br>0.505 µg/ml (LPV)
[9]	HPTLC - densitometric Scanner	Dosage form, biological fluids, plasma & urine, Degradation	Ethyl acetate<br>Methanol<br>Ethanol<br>Acetone	Silica gel 60 F254, mobile phase ethyl acetate: methanol: water: glacial acetic acid (30: 1.5: 1: 0.2%).	2 - 12 µg/band (SBV)<br>0.45 - 6 µg/band (LPV)	0.6 µg/band (SBV)<br>0.1 µg/band (LPV)	1.9 µg/band (SBV)<br>0.33 µg/band (LPV)
[10]	RP - HPLC method by UV and fluorescence detectors	Tablet Dosage Form	Methanol<br>Acetonitrile	C18 column, mobile phase ACN:methanol:0.01% TEA (35: 35:30). Flow rate 1.2 mL/min. UV 261 nm switched online at 5 min	1 - 40 µg/mL (SBV)<br>0.4 - 20 µg/mL (LPV)	0.012 µg/ml (SBV)<br>0.011 µg/ml (LPV)	0.040 µg/ml (SBV)<br>0.036 µg/ml (LPV)

				to 333 nm. FL<br>Excitation/emission:<br>333 nm/404 nm (LPV)			
[11]	RP - HPLC	Tablet Dosage Form	Methanol	Pentafluorophenyl (PFP) column, gradient mobile phase water (A): Methanol (B). Flow rate 1ml/min. UV 260 nm switched at 5 min to 330 nm	5 - 80 µg/ml (SBV)<br>2 - 50 µg/mL (LPV)	1.25 µg/ml (SBV)<br>0.50 µg/ml (LPV)	4.00 µg/ml (SBV)<br>2.00 µg/ml (LPV)
[12]	RP – HPLC<br><br>UV SPECTROSCOPY	Tablet Dosage Form	Methanol<br>Acetonitrile	UV Methods:<br>Q - Absorbance ratio and dual wavelength method.<br>HPLC:<br>C18 column, mobile phase ACN: 0.1% TFA (32:68 %v/v). Flow rate 1ml/min. Detection 245 nm.	UV:<br>10 - 30 µg/ml (SBV)<br>2.25 - 6.75 µg/ml (LPV)<br>HPLC:<br>100 - 600 µg/ml (SBV)<br>22.5 - 135 µg/ml (LPV)	0.139 µg/ml (SBV)<br>0.094 µg/ml (LPV)	0.412 µg/ml (SBV)<br>0.284 µg/ml (LPV)
[13]	LC-MS	Biological fluids, Plasma & Liver	Methanol<br>Acetonitrile	C18 column, gradient mobile phase Methanol (A): 0.1% formic acid (B). Flow rate 0.4ml/min.	10 - 2000 ng/mL<br>25 - 2000 ng/mL	3.12 ng/mL (SBV)<br>4.36 ng/mL (LPV)	10.40 ng/mL (SBV)<br>14.53 ng/mL (LPV)
[14]	HPTLC - densitometric Scanner	Rabbit plasma	Methanol<br>Ethyl acetate	Silica gel 60 F254, mobile phase ethyl acetate: glacial acetic acid (100:5)	40 - 640 ng/band (SBV)<br>9 - 144 ng/band (LPV)	10.61 ng/band (SBV)<br>2.54 ng/band (LPV)	32.14 ng/band (SBV)<br>7.70 ng/band (LPV)
[15]	UPLC - MS/MS	Drugs & metabolite in plasma.	Methanol<br>Acetonitrile	C18 column. Gradient mobile phase ACN (A): 0.1% formic acid (B). Flow rate 0.4ml/min.	10 - 2000 ng/mL (SBV)<br>2 - 500 ng/mL (LPV)	- -	10 ng/mL (SBV)<br>2 ng/mL (LPV)
[16]	UPLC - UV	Human Plasma	Acetonitrile<br>Diethyl ether<br>Ethanol<br>Methanol	C18 column, mobile phase 0.1% formic acid: ACN (60:40), Flow rate 0.5ml/min. UV 260 and 328 nm (SBV & LPV).	20–1280 ng/mL (SBV)<br>5–1280 ng/mL (LPV)	- -	20 ng/mL (SBV)<br>5 ng/mL (LPV)
[17]	UV spectrophotometric	Tablet Dosage Form	Methanol	Zero order at 333nm (LPV)<br>For (SBV)<br>228 nm and 270 nm to obtain the ratio spectrum, then First Derivative, Ratio Subtraction, Mean Centering	7.5 - 90 µg/ml (SBV)<br>2.5 - 30 µg/ml (LPV)	0.91 µg/ml (SBV)<br>0.28 µg/ml (LPV)	2.77 µg/ml (SBV)<br>0.85 µg/ml (LPV)
[18]	HPTLC - densitometric Scanner<br><br>RP - HPLC	Tablet Dosage Form	Ethanol<br>Toluene	HPTLC:<br>Silica gel 60 F254, mobile phase toluene: ethanol: ammonia (4:1:0.2)<br>HPLC:<br>C18 column, mobile phase phosphate buffer: ethanol (60:40). Flow rate 1ml/min. UV 265nm.	HPTLC:<br>0.8 - 25.6 µg/band (SBV)<br>0.4 - 12.8 µg/band (LPV)<br>HPLC:<br>6 - 100 µg/mL (SBV)<br>4 - 80 µg/mL (LPV)	HPTLC:<br>0.21 µg/band (SBV)<br>0.1 µg/band (LPV)<br>HPLC:<br>1.88 µg/ml (SBV)<br>1.13 µg/ml (LPV)	HPTLC:<br>0.63 µg/band (SBV)<br>0.31 µg/band (LPV)<br>HPLC:<br>5.69 µg/ml (SBV)<br>3.42 µg/ml (LPV)
[19]	UV spectrophotometric	Tablet Dosage Form	Methanol	1st derivative for both Isosbestic point (SBV), Absorbance ratio (SBV).	2 - 50 ug/ml<br>(SBV & LPV)	0.08 µg/ml (SBV)<br>0.11 µg/ml (LPV)	0.23 µg/ml (SBV)<br>0.33 µg/ml (LPV)
[20]	RP - HPLC	Tablet Dosage Form & Human Plasma	Acetonitrile	C18 column, mobile phase gradient mixture of acetonitrile:	1 - 45 µg/mL<br>(SBV & LPV)	- -	- -

				water. UV 260nm (SBV) & 330nm (LPV).			
[21]	UV spectrophotometric<br>HPTLC<br>RP - HPLC	Tablet Dosage Form	Methanol Chloroform	UV:<br>Zero-crossing derivative, ratio spectra derivative & ratio difference spectrophotometry<br>HPTLC:<br>silica gel 60 F254, mobile phase chloroform and methanol (94:6)<br>HPLC:<br>C8 column, mobile phase phosphate buffer: methanol (20:80), flow rate 1.2ml/min. UV 262nm (SBV) 332nm (LPV)	UV:<br>6 - 60 µg/ml (SBV)<br>4 - 40 µg/ml (LPV)<br>HPTLC:<br>20 - 150 µg/ml (SBV)<br>10 - 120 µg/ml (LPV)<br>HPLC:<br>4 - 400 µg/ml (SBV)<br>1 - 100 µg/ml (LPV)	UV:<br>0.42 µg/ml (SBV)<br>0.55 µg/ml (LPV)<br>HPTLC:<br>5.74 µg/ml (SBV)<br>3.23 µg/ml (LPV)<br>HPLC:<br>0.80 µg/ml (SBV)<br>0.20 µg/ml (LPV)	UV:<br>1.24 µg/ml (SBV)<br>1.67 µg/ml (LPV)<br>HPTLC:<br>17.41 µg/ml (SBV)<br>9.79 µg/ml (LPV)<br>HPLC:<br>2.67 µg/ml (SBV)<br>0.67 µg/ml (LPV)
[22]	RP - HPLC	Tablet Dosage Form	Methanol Acetonitrile	C18 column. Mobile phase TFA: ACN: Methanol (30:50:20%). Flow rate 1ml/min. UV 267nm.	40 - 120 µg/ml (SBV)<br>10 - 30 µg/ml (LPV)	0.015 µg/ml (SBV)<br>0.012 µg/ml (LPV)	0.05 µg/ml (SBV)<br>0.042 µg/ml (LPV)
[23]	RP - HPLC	Impurities, tablet dosage form, dissolution.	Acetonitrile Methanol	C8 column, mobile phase gradient phosphate buffer: Methanol. Flow rate 1.5 ml/min. UV 260nm& 332nm (SBV & LPV).	2 - 300 µg/ml (SBV)<br>0.5 - 75 µg/ml (LPV)	0.2 µg/ml (SBV)<br>0.1 µg/ml (LPV)	0.67 µg/ml (SBV)<br>0.33 µg/ml (LPV)
[24]	LC-MS/MS	Metabolite, Human Plasma	Methanol Acetonitrile Ethyl Acetate	C_8 column, mobile phase gradient ammonium formate: ACN: Methanol. Flow rate 0.7ml/min.	0.3 - 3000 ng/ml (SBV)<br>0.1 - 1000 ng/ml (LPV)	- -	0.3 ng/ml (SBV)<br>0.1 ng/ml (LPV)
[25]	RP - HPLC	Tablet Dosage Form	Acetonitrile Methanol	C_8 column, mobile phase 0.1% OPA: ACN (60:40). Flow rate 1 ml/min. UV 260nm. 30°C	40 - 240 µg/ml (SBV)<br>9 - 54 µg/ml (LPV)	0.67 µg/ml (SBV)<br>0.70 µg/ml (LPV)	2.02 µg/ml (SBV)<br>2.12 µg/ml (LPV)
[26]	RP - HPLC	Tablet Dosage Form & Stability	Acetonitrile	C18 column, mobile phase 0.1% OPA & ACN (45:55). Flow rate 1ml/min. UV 270nm.	100 - 600 µg/ml (SBV)<br>22.5 - 135 µg/ml (LPV)	0.22 µg/ml (SBV)<br>0.061 µg/ml (LPV)	0.65 µg/ml (SBV)<br>0.186 µg/ml (LPV)
[27]	UPLC - UV	Tablet Dosage Form & Stability	Acetonitrile	C18 column, mobile phase phosphate buffer: ACN (50:50) Flow rate 0.3 ml/min. UV 220nm.	100 - 600 µg/ml (SBV)<br>22.5 - 135 µg/ml (LPV)	1.29 µg/ml (SBV)<br>0.31 µg/ml (LPV)	3.91 µg/ml (SBV)<br>0.95 µg/ml (LPV)
[28]	UV spectrophotometric	Tablet Dosage Form	Methanol	zero order at 333nm (LPV)<br>1st derivative, ratio difference & ratio subtraction (SBV)	5 - 50 ug/ml<br>(SBV & LPV)	0.87 µg/ml (SBV)<br>0.91 µg/ml (LPV)	2.63 µg/ml (SBV)<br>2.76 µg/ml (LPV)
[29]	UPLC - MS/MS	Human plasma	Acetonitrile Dimethyl sulfoxide Methanol	C18 column, gradient mobile phase ammoniumformate with 0.005% formic acid in water (A): ammoniumformate with 0.005% formic acid in methanol (B) and ammoniumformate with 0.005% formic acid in methanol: acetonitrile;10:90 (C). Flow rate 0.4 mL/min. 40 °C.	5.0 - 2500 µg/L (SBV)<br>7.5 - 1500 µg/L (LPV)	- -	5.0 µg/L (SBV)<br>7.8 µg/L (LPV)
[30]	RP - HPLC	Tablet Dosage Form	Acetonitrile Methanol	C18 column, mobile phase phosphate buffer: Methanol	20 -100 µg/ml (SBV)<br>10 - 50µg/ml (LPV)	3.04 µg/ml (SBV)<br>2.95 µg/ml (LPV)	10 µg/ml (SBV)<br>9.8 µg/ml (LPV)

				(30:70). Flow rate 1ml/min. UV 270nm.			
[31]	RP - HPLC	Tablet Dosage Form & Stability	Methanol	C18 column, mobile phase Methanol: 0.05% acidic acid Water (83:17). Flow rate 1ml/min. UV 245nm.	40-200µg/ml (SBV)<br>10-50µg/ml (LPV)	3.36 µg/ml (SBV)<br>1.09 µg/ml (LPV)	10.19 µg/ml (SBV)<br>3.30 µg/ml (LPV)
[32]	RP-HPLC and Spectroscopic Method (Chemometric)	Tablet Dosage Form	Methanol<br>Acetonitrile	UV:<br>1st derivative<br>HPLC:<br>C18 column, mobile phase Methanol: Acetonitrile: 1% Ammonium acetate (50:20:30) Flow rate 1ml/min. UV 281nm.	UV:<br>8-48 µg/ml (SBV)<br>2-12 µg/ml (LPV)<br>HPLC:<br>4-44 µg/ml (SBV)<br>1-11 µg/ml (LPV)	UV:<br>0.209 µg/ml (SBV)<br>0.0710 µg/ml (LPV)<br>HPLC:<br>0.0707 µg/ml (SBV)<br>0.328 µg/ml (LPV)	UV:<br>0.633 µg/ml (SBV)<br>0.2153 µg/ml (LPV)<br>HPLC:<br>0.2144 µg/ml (SBV)<br>0.995 µg/ml (LPV)
[33]	RP - HPLC	Tablet Dosage Form & Stability	Acetonitrile	C18 column, mobile phase ACN: 0.1% TFA (30:70). Flow rate 1ml/min. UV 245nm.	100-600 µg/ml (SBV)<br>22.5-135 µg/ml (LPV)	0.395 µg/ml (SBV)<br>0.132 µg/ml (LPV)	1.197 µg/ml (SBV)<br>0.401 µg/ml (LPV)
[34]	UPLC–MS/MS	Fasting & fed bioequivalence Studies. Human Plasma	Methanol<br>Acetonitrile	C_{18} column, mobile phase 0.1% formic acid: ACN (50:50). Flow rate 0.4 ml/min.	0.25–3500 ng/ml (SBV)<br>5–2000 ng/ml (LPV)	--	0.25 ng/ml (SBV)<br>5 ng/ml (LPV)
[35]	UV spectrophotometric	Tablet Dosage Form	Methanol	Simultaneous equation (Verdot's) method 260 & 296nm (SBV & LPV)	5-25 µg/ml<br>(SBV & LPV)	0.00818µg/ml (SBV)<br>0.02112 µg/ml (LPV)	0.136502µg/ml(SBV)<br>0.07041 µg/ml (LPV)
[36]	RP-HPLC<br>(QbD)	Tablet Dosage Form & Stability	Methanol	C18 column, mobile phase methanol: 0.01M ammonium acetate (70:30). flow rate 1ml/min. UV 254nm.	50-150 µg/ml (SBV)<br>11-33 µg/ml (LPV)	0.45 µg/ml (SBV)<br>3.85 µg/ml (LPV)	1.5 µg/ml (SBV)<br>11 µg/ml (LPV)
[37]	RP - HPLC	Tablet Dosage Form	Methanol<br>Acetonitrile	C8 column, gradient mobile phase ACN: phosphate buffer. Flow rate 2ml/min. UV 260nm & 330nm (SBV & LPV). 40 °C.	1.0–20.0 µg/ml<br>(SBV & LPV)	0.034 µg/ml (SBV)<br>0.039 µg/ml (LPV)	0.103 µg/ml (SBV)<br>0.118 µg/ml (LPV)
[38]	UHPLC–MS/MS	Human Plasma	Acetonitrile<br>Methanol<br>DMSO	C18 column. Gradient mobile phase ammonium acetate (A) and ACN (B). Flow rate 0.4 mL/min. 50 °C.	80-2000 ng/ml<br>(SBV & LPV)	0.7 ng/ml (SBV)<br>5.9 ng/ml (LPV)	11.7 ng/ml (SBV & LPV)
[39]	UV spectrophotometric	Tablet Dosage Form	Methanol	Simultaneous equation method 237nm & 247nm (SBV & LPV) & 1st derivative.	20-120 µg/ml (SBV)<br>4.5-27 µg/ml (LPV)	0.5µg/ml (SBV)<br>1 µg/ml (LPV)	2 µg/ml (SBV)<br>3.5 µg/ml (LPV)
[40]	UV spectrophotometric	Tablet Dosage Form	Methanol	Zero order 333 nm (LPV).<br>1st derivative 247.2 & 260.8 nm (SBV & Paracetamol)	8–60 µg/mL (SBV)<br>4–22 µg/mL (LPV)	-	-
[41]	LC-MS/MS	Human Plasma & Pharmacokinetics	Acetonitrile<br>Methanol<br>Diethyl ether<br>Dichloromethane	Mobile phase Ammonium formate: ACN (10: 90). flow rate 0.55ml/min.	0.1-2000ng/ml (SBV)<br>0.1-600 ng/ml (LPV)	-	0.1 ng/ml (SBV & LPV)
[42]	UV spectrophotometric (Chemometric)	Tablet Dosage Form	Methanol	classical least squares (CLS), principal component regression (PCR), simultaneous equation, and amplitude modulation	4-40 µg/mL (SBV)<br>0.9-9 µg/mL (LPV)	0.479 µg/mL (SBV)<br>0.168 µg/mL (LPV)	1.454 µg/mL (SBV)<br>0.509 µg/mL (LPV)

[43]	UHPLC-UV	Human plasma	Acetonitrile<br>Methanol	C18 column, mobile phase ACN, and 0.1% OPA (42:58) . Flow rate 0.4ml/min. UV 254nm.	(5–60) µg/mL (SBV)<br>(2–22) µg/mL (LPV)	0.153 µg/mL (SBV)<br>0.074 µg/mL (LPV)	0.464 µg/mL (SBV)<br>0.225 µg/mL (LPV)
[44]	UHPLC-UV<br>(Chemometric)	Tablet Dosage Form	Acetonitrile	C18 column, mobile phase ACN: Sodium perchlorate (40: 60). flow rate 1.5ml/min. UV 210nm.35°C.	162.40-243.71 µg/mL (SBV)<br>35.95–54.12 µg/mL (LPV)	0.1 µg/mL (SBV)<br>0.05 µg/mL (LPV)	162.40 µg/mL (SBV)<br>35.95 µg/mL (LPV)
[45]	HPTLC - densitometric Scanner<br><br>HPTLC-MS	Tablet Dosage Form & degradation	Ethanol<br>Ethyl acetate	Densitometric:<br>Silica gel F_{254} plates, mobile phase ethyl acetate: water: ethanol (94:5:1).<br>MS:<br>degradation products	100–5000 ng/spot (SBV)<br>100–3000 ng/spot (LPV)	30 ng/spot (SBV)<br>29 ng/spot (LPV)	90 ng/spot (SBV)<br>88 ng/spot (LPV)
[46]	RP-HPLC<br>(QbD)	Tablet Dosage Form & degradation	Methanol<br>Heptane-1-sulphonic acid<br>Diethyl ether	Promosil CN column, gradient mobile phase methanol: 0.005M Heptane-1-sulphonic acid. Flow rate 1ml/min. UV 220nm and changed to 261nm (4.5min), then 324nm (9 min)	5–500 µg/mL (SBV)<br>1–112.5 µg/mL (LPV)	1.351 µg/mL (SBV)<br>4.093 µg/mL (LPV)	0.241 µg/mL (SBV)<br>0.729 µg/mL (LPV)
[47]	RP-HPLC	Tablet Dosage Form & Degradation	Methanol	C8 column, Mobile Phase: 0.1% OPA: Methanol (45:55). Flow rate 1ml/min. UV 238nm. 30°C.	50-150 µg/ml<br>(SBV & LPV)	--	--
[48]	HPTLC - densitometric Scanner	Tablet Dosage Form	Chloroform<br>Acetone<br>Toluene<br>Ethyl acetate<br>n-Hexane<br>Methanol	Silica gel 60 F254, mobile phase mixture of ethyl acetate: methanol: toluene: acetone: acetic acid (2:1.5:4.5:2.0:0.2).	200-1200 ng/spot (SBV)<br>45-270 ng/spot (LPV)	5.3411 ng/spot (SBV)<br>0.6598 ng/spot (LPV)	16.1853 ng/spot (SBV)<br>1.999 ng/spot (LPV)
[49]	RP-HPLC	Tablet Dosage Form & stability	Acetonitrile	C18 column, mobile phase ACN: 0.1% OPA (35:65). Flow rate 1ml/min. 30°C.	100 - 600 µg/mL (SBV)<br>22.5 - 135 µg/mL (LPV)	1.22 µg/mL (SBV)<br>0.184 µg/mL (LPV)	3.7 µg/mL (SBV)<br>0.557 µg/mL (LPV)
[50]	RP-HPLC	Tablet Dosage Form	Acetonitrile<br>Methanol	C18 column, mobile phase Acetonitrile: OPA (80: 20). Flow rate 0.9ml/min	20-100 µg/mL (SBV)<br>5-25 µg/mL (LPV)	--	--
[51]	^{1}H and ^{19}F NMR	Tablet Dosage Form	Dimethyl sulfoxide	Relaxation delay 10s. Exponential line-broadening 0.3Hz. Acquisition time 4.08 and 0.73s. Sweep width, 8012.820 and 89285.711Hz; data points, 65536 and 131072 for 1H-NMR and 19F-NMR, correspondingly	2–24 mg/mL<br>(SBV & LPV)	0.387mg/mL (SBV)<br>0.275mg/mL (LPV)	1.289 mg/mL (SBV)<br>0.915 mg/mL (LPV)
[52]	RP-HPLC	Human plasma	Acetonitrile<br>Methanol<br>Diethyl ether<br>Dichloromethane<br>Methyl t-butyl ether	C18 column, mobile phase Acetate buffer: Methanol: ACN (20:60:20). Flow rate 1ml/min. UV 238nm.	250-2000 ng/ml<br>(SBV & LPV)	--	200ng/ml<br>(SBV & LPV)
[53]	RP-HPLC	Stability	Methanol	C8 column, mobile phase 0.1% OPA: Methanol (45:55). Flow rate 1ml/min. UV 238nm. 30˚C	200-600 µg/ml (SBV)<br>45-135 µg/mL (LPV)	--	2.2063 µg/ml (SBV)<br>0.695 µg/mL (LPV)

[54]	UV spectrophotometer (QbD & Chemometric)	Tablet Dosage Form	Methanol	PLS, GA-PLS, ANN, GA-ANN	-	-	-
[55]	HPTLC - densitometric Scanner	Tablet Dosage Form	Methanol<br>Ethyl acetate<br>Hexane<br>Chloroform	60 F254 silica gel plates. mobile phase ethyl acetate: hexane: TEA (90:9:1)	4-24 ug/band (SBV)<br>0.9-5.4 ug/band (LPV)	0.822 ug/band (SBV)<br>0.138ug/band (LPV)	2.491ug/band (SBV)<br>0.417 ug/band (LPV)
[56]	UV spectrophotometer<br><br>HPTLC<br><br>HPLC	Tablet Dosage Form & Stability	Methanol<br>Acetonitrile<br>Toluene<br>Chloroform<br>Ethyl acetate<br>Hexane	UV:<br>Simultaneous equation & 2nd derivative<br>HPTLC:<br>silica gel G60 F254. Mobile phase Hexane: Ethyl acetate: methanol (5:3:2)<br>HPLC:<br>C18 column. Mobile phase 0.1%OPA: ACN (30:70). Flow rate 0.8ml/min. UV 254nm.	UV:<br>10-70 µg/ml (SBV)<br>10-40 µg/ml (LPV)<br>HPTLC:<br>440-3080 ng/spot(SBV)<br>100-700 ng/spot (LPV)<br>HPLC:<br>1-10 µg/ml (SBV &LPV)	UV:<br>0.0044 µg/ml<br>(SBV & LPV)<br>HPTLC:<br>0.4345ng/spot(SBV)<br>0.13ng/spot (LPV)<br>HPLC:<br>0.0006 µg/ml (SBV)<br>0.008 µg/ml (LPV)	UV:<br>0.0134 µg/ml<br>(SBV & LPV)<br>HPTLC:<br>1.316ng/spot(SBV)<br>0.40ng/spot (LPV)<br>HPLC:<br>0.0020 µg/ml (SBV)<br>0.27 µg/ml (LPV)
[57]	RP-HPLC	Tablet Dosage Form	Methanol	Luna PFP column. Mobile phase gradient water (A): methanol (B). flow rate 1ml/min. UV 260nm.	5-80 µg/ml (SBV)<br>2-50 µg/ml (LPV)	1.25 µg/ml (SBV)<br>0.5 µg/ml (LPV)	4 µg/ml (SBV)<br>2 µg/ml (LPV)
[58]	LC-MS	Rat plasma & pharmacokinetics	Acetonitrile<br>Methanol	C18 column. Mobile phase gradient 0.1%formic acid (A): ACN & methanol 1:1 (B). flow rate 0.4ml/min.	4–2000 ng/ml (SBV)<br>1–1000 ng/ml (LPV)	--	42000 ng/ml (SBV)<br>1 ng/ml (LPV)
[59]	UV spectrophotometer (Chemometric)	Tablet Dosage Form	Acetonitrile	least squares support vector machine (LS-SVM) and artificial neural networks (ANNs)	24–64 ppm (SBV)<br>6–16 ppm (LPV)	-	-
[60]	HPTLC<br><br>RP-HPLC	Tablet Dosage Form & Stability	Toluene<br>Methanol<br>Ethyl acetate<br>Acetonitrile	HPTLC:<br>Silica gel 60 F254. Mobile phase toluene: methanol: ethyl acetate: acetic acid (6 : 2 : 2 : 0.3).<br>HPLC:<br>C18 column. Mobile phase Phosphate buffer: ACN: Methanol (60:30:10). Flow rate 1.5ml/min. UV 254nm.	HPTLC:<br>1000-6000 ng/spot (SBV)<br>100-600 ng/spot (LPV)<br>HPLC:<br>5-60 µg/ml (SBV)<br>2-12 µg/ml (LPV)	HPTLC:<br>0.298 ng/spot (SBV)<br>0.238 ng/spot (LPV)<br>HPLC:<br>0.348 µg/ml (SBV)<br>0.279 µg/ml (LPV)	HPTLC:<br>0.852 ng/spot (SBV)<br>0.997 ng/spot (LPV)<br>HPLC:<br>1.052 µg/ml (SBV)<br>0.789 µg/ml (LPV)
[61]	Micellar liquid chromatography (QbD)	Tablet Dosage Form	Methanol<br>Triethylamine<br>Isopropanol	C18 column. Mobile phase SDS: isopropanol: TEA (79.7:20:0.3). flow rate 1.5ml/min. UV 210nm. 30°C	10–100 µg/ml (SBV)<br>20–200 µg/ml (LPV)	3.3 µg/ml (SBV)<br>6.6 µg/ml (LPV)	10 µg/ml (SBV)<br>20 µg/ml (LPV)
[62]	Capillary Electrophoresis	Tablet Dosage Form	Methanol<br>Ethanol<br>Isopropanol<br>Acetonitrile	Fused silica capillary. Acetate buffer (ground electrolyte). Running potential 25 kV. Hydrodynamic injection 5s. 70mbar pressure	5-600 µg/ml (SBV)<br>20-400 µg/ml (LPV)	1.5 µg/ml (SBV)<br>6 µg/ml (LPV)	5 µg/ml (SBV)<br>20 µg/ml (LPV)

[63]	Micellar Capillary Electrophoresis	Tablet Dosage Form	Methanol<br>Acetonitrile	Fused silica-capillaries. The background electrolyte comprises SDS + 20%ACN in a disodium tetraborate buffer. The applied voltage is +30 kV. Hydrodynamic injection 50s. pressure 50mbar	1.25–40 µg/ml (SBV)<br>2.50–40 µg/ml (LPV)	0.63 µg/ml (SBV)<br>1.3 µg/ml (LPV)	1.3 µg/ml (SBV)<br>2.5 µg/ml (LPV)
[64]	HPTLC - densitometric Scanner	Tablet Dosage Form	Ethyl acetate<br>Hexane<br>Hexane sulfonate<br>Methanol<br>Ethanol	silica gel 60 F254. Mobile phase Ethyl acetate: Hexane: Methanol (8:1.25:0.75).	45–3600ng/band (SBV)<br>60–1980ng/band (LPV)	13 ng/band (SBV)<br>16.5 ng/band (LPV)	39.5 ng/band (SBV)<br>50 ng/band (LPV)
[65]	Voltammetry (Electrochemical impedance spectroscopy)	Tablet Dosage Form & Human Plasma	--	Modifying a glassy carbon surface with layers of multi-walled carbon nanotube, ionic liquid crystal (ILC), graphene (RGO), and MnO2. AC frequency range of 100 kHz to 0.1 Hz	0.2–150 µmol.dm^{-3} (SBV)<br>0.007–15 µmol.dm^{-3} (LPV)	9.8 nmol.dm^{-3} (SBV)<br>0.11 nmol.dm^{-3} (LPV)	0.39 nmol.dm^{-3} (LPV)
[66]	Voltammetry (Electrochemical impedance spectroscopy)	Tablet Dosage Form & Human Plasma	--	Multi-walled carbon nanotubes were placed on a glassy carbon electrode surface, followed by electrodeposition of cobalt nanoparticles. Impedance is applied in the range of 0.1 100 kHz. Excitation signal 10mV amplitude	[illegible].3–8 & 10–100 umol/L (SBV)<br>0.02–1 & 3–100 umol/L (LPV)	7.29 nmol/L (SBV)<br>0.277 nmol/L (LPV)	24.3 nmol/L (SBV)<br>0.923 nmol/L (LPV)
[67]	RP-HPLC	Tablet Dosage Form	Ethanol<br>Methanol	Core-shell and totally porous particle stationary phases. Gradient mobile phosphate buffer & sodium octane sulfonate (A): Ethanol (B). Flow rate 0.3ml/min. UV 210nm. 35°C.	2–200 µg/mL (SBV &LPV)	50 ng/ml (SBV)<br>200 ng/ml (LPV)	0.2 ug/ml (SBV)<br>0.7 ug/ml (LPV)
[68]	RP-HPLC	River water	Chloroform<br>Methanol<br>Acetonitrile	C18 column. Mobile phase phosphate buffer: ACN (50:50). Flow rate 1ml/min. UV 220nm. 30 °C	0.01–5.0 µg/mL (SBV & LPV)	0.006 µg/ml (SBV)<br>0.012 µg/ml (LPV)	0.02 µg/ml (SBV)<br>0.04 µg/ml (LPV)
[69]	RP-HPLC<br><br>LC-MS	Generic and Brand Products. Dissolution and Human Plasma	Acetonitrile<br>Methanol	In vitro:<br>C18 column. Mobile phase ACN: phosphate buffer (60:40). Flow rate 1.5ml/min. UV 260nm.<br>In vivo:<br>C18 column. Mobile phase ACN: 0.1% formic acid (50:50). Flow rate 0.4ml/min. 35 °C	In vitro:<br>8.8–61.6 ug/mL (SBV)<br>2–14 ug/mL (LPV)<br>In vivo:<br>Same as [34]	-	-
[70]	RP-HPLC	Tablet Dosage Form & Human Plasma	Acetonitrile<br>Diethyl ether	C18 Column. Mobile phase 0.1%TEA: ACN (70:30). Flow rate 0.8ml/min. 210 & 300nm (SBV & LPV)	0.4–20 µg/mL (SBV & LPV)	0.04 µg/mL (SBV)<br>0.01 µg/ml (LPV)	0.15 µg/mL (SBV)<br>0.03 µg/ml (LPV)

[71]	RP-HPLC	Tablet Dosage Form	Methanol	C18 Column. Mobile phase Phosphate buffer: Methanol (45:55). Flow rate 1ml/min. UV 259nm. 35 °C	5-25 µg/ml (SBV)<br>2-10 µg/ml (LPV)	0.24 µg/ml (SBV)<br>0.06 µg/ml (LPV)	0.73 µg/ml (SBV)<br>0.19 µg/ml (LPV)
[72]	RP-HPLC	Tablet Dosage Form	Acetonitrile<br>Methanol	C18 Column. Mobile phase ACN: Methanol (60:40). Flow rate 1ml/min. UV 254nm.	20-80 ppm<br>(SBV & LPV)	-	-
[73]	RP-HPLC<br>(QbD)	Tablet Dosage Form	Acetonitrile<br>Methanol	C18 column. Mobile phase phosphate buffer: ACN (50:50). Flow rate 1ml/min. UV 260nm.	60–360 µg/mL (SBV)<br>40–140 µg/mL (LPV)	1.389 µg/mL (SBV)<br>0.622 µg/mL (LPV)	4.167 µg/mL (SBV)<br>1.866 µg/mL (LPV)
[74]	RP-HPLC	Tablet dosage form and degradation	Methanol	C18 column. Mobile phase Methanol: Water(70:30). Flow rate 0.6ml/min. UV 235nm.	32–48 µg/mL (SBV)<br>7.2–10.8 µg/mL (LPV)	0.341 µg/mL (SBV)<br>0.208 µg/mL (LPV)	1.034 µg/mL (SBV)<br>0.631 µg/mL (LPV)
[75]	RP-HPLC	Tablet Dosage Form	Acetonitrile<br>Methanol	C18 column. Mobile phase 0.1% OPA: ACN (55:45). Flow rate 1ml/min. UV 230nm.	100-600 µg/mL (SBV)<br>22.5-135 µg/mL (LPV)	0.02µg/ml (SBV)<br>0.28µg/ml (LPV)	0.07 µg/ml (SBV)<br>0.85µg/ml (LPV)
[76]	HPTLC - densitometric Scanner	Tablet dosage form and degradation	Hexane<br>Ethyl acetate<br>Methanol	Silica gel 60F254. Mobile phase hexane: ethyl acetate: methanol (5:3:2) with 3 drops of ammonia.	440-3080 ng/spot (SBV)<br>100-700 ng/spot (LPV)	0.4345 ng/spot (SBV)<br>0.13 ng/spot (LPV)	1.316 ng/spot (SBV)<br>0.40 ng/spot (LPV)
[77]	UV spectrophotometer<br><br>RP-HPLC	Tablet dosage form	Methanol<br>Triethanolamine	UV:<br>1st derivative<br>HPLC<br>C18 column. Mobile phase Phosphate buffer: Methanol: TEA (40:60:0.1). flow rate 1ml/min. UV 240nm.	UV & HPLC:<br>20-60 µg/mL (SBV)<br>4.5-13.5 µg/mL (LPV)	5.703 µg/mL (SBV)<br>0.33 µg/mL (LPV)	17.28 µg/mL (SBV)<br>0.99 µg/mL (LPV)
[78]	RP-HPLC	Tablet dosage form and degradation	Acetonitrile	C18 column. Mobile phase phosphate buffer: ACN (60:40). Flow rate 0.8ml/min. UV 282nm. 30 °C.	80-240 µg/mL (SBV)<br>18-54 µg/mL (LPV)	0.225 µg/mL (SBV)<br>0.074 µg/mL (LPV)	0.751 µg/mL (SBV)<br>0.249 µg/mL (LPV)
[79]	RP-HPLC	Tablet dosage form	Acetonitrile	C18 column. Mobile phase phosphate buffer: ACN (55:45). Flow rate 1ml/min. UV 213nm.	50-175 µg /ml<br>(SBV & LPV)	-	-
[80]	Spectrofluorimetric method	LPV in synthetic mixture with SBV	Methanol<br>Acetonitrile<br>Ethanol<br>Isopropanol<br>Acetone<br>Dimethyl formamide<br>DMSO	332/387 nm (Excitation/Emission) for LPV	6 - 120 ng/mL (LPV)	1.939 ng/mL (LPV)	5.875 ng/mL (LPV)
[81]	Spectrofluorimetric method (Micellar-based)	Dosage form & Human plasma	Acetone<br>Methanol<br>Acetonitrile<br>1,4 Dioxan<br>Dimethylsulphoxide<br>Ethanol<br>Dimethylformamide	340nm and 425nm (Excitation/Emission) for LPV	0.1 - 2.0 µg/ml (LPV)	0.028 µg/ml (LPV)	0.084 µg/ml (LPV)
[82]	Spectrofluorimetric method	Tablet Dosage Form	Acetonitrile<br>Chloroform	340 & 405nm (Excitation & Emission) for LPV	100–800 ng/ml (LPV)	16.8 ng/ml (LPV)	50.9 ng/ml (LPV)

			Ethanol<br>Methanol				
[83]	Spectrofluorimetric method (Micellar-based)	Dosage form & Human plasma	Methanol<br>Ethanol<br>Acetonitrile<br>Dimethyl formamide	Synchronous $\Delta\lambda$ = 120 nm	36–540 ng/ml (LPV)	6 ng/ml (LPV)	17 ng/ml (LPV)
[84]	Spectrofluorimetric method	Dosage form, Human plasma & Degradation	Methanol<br>Ethanol<br>Isopropanol<br>Acetonitrile	340 & 430nm (Excitation & Emission) for LPV	1–400 ng/ml (LPV)	0.25 ng/ml (LPV)	1.10 ng/ml (LPV)
[85]	Spectrofluorimetric method	Biological fluids, pharmacokinetic study, content uniformity	Acetone<br>Chloroform<br>Dichloromethane<br>Ethyl acetate<br>Ethanol<br>Hexane<br>Methanol<br>Polyethylene glycol<br>Acetonitrile	321&375nm (Excitation & Emission) for LPV	5–150 ng/ml (LPV)	0.9 ng/ml (LPV)	2.7 ng/ml (LPV)
[86]	Voltammetry	Tablet, human urine, plasma.	-	Zeolite modified carbon paste electrode. Square wave.	5.03×10^{-8} -1×10^{-4} M (LPV)	7.5×10^{-9} M (LPV)	25×10^{-9} M (LPV)
[87]	Voltammetry	Tablet Dosage Form	Ethanol	Boron-doped diamond electrode. Cyclic voltammetry.	0.5–60.0 µg/mL (LPV)	0.12 µg/mL (LPV)	0.4 µg/mL (LPV)
[88]	Voltammetry	Tablet Dosage Form & Rat plasma	Methanol<br>Ethanol<br>Acetonitrile<br>Dimethylformamide<br>Tetrahydrofuran<br>1,4 Dioxane	MnO2-modified graphite electrode. Square wave & cyclic voltammetry.	0.025–3.60 µmol/L (LPV)	4.5 ng/ml (LPV)	16.2 ng/ml (LPV)
[89]	Voltammetry	Tablet dosage form, urine, and degradation.	Dimethyl sulfoxide (DMSO)<br>Methanol	The glassy carbon electrode was modified with functionalized multi-walled carbon nanotubes and reduced graphene oxide nanocomposite. Differential pulse voltammetry.	0.53–74.13 ng/mL (SBV)	0.05 ng/mL (SBV)	-
[90]	Voltammetry	Tablet Dosage Form & Human Plasma	Methanol	3D spinel ferromagnetic NiFe2O4 nanospheres and reduced graphene oxide supported by morpholinium acid sulphate as an ionic liquid. Differential pulse voltammetry	0.4–350.0 ng/mL (LPV)	0.133 ng/mL (LPV)	-
[91]	Voltammetry	Human plasma	Methanol	MIP was obtained by polymerizing p-aminothiophenol on N, S co-doped graphene quantum dots in the presence of gold nanoparticles to form a gold-sulfur covalent network. Differential pulse voltammetry	1–400 nM (SBV)	0.36 nM (SBV)	-

[92]	Voltammetry	Tablet Dosage Form & Human Plasma	Methanol	polymethyl dopa polymer electro grafted onto a pencil graphite electrode in the presence of SBV as a template. Differential pulse voltammetry	1×10^{-11} -1×10^{-13} M (SBV)	3.1×10^{-14} M (SBV)	9.39×10^{-14} M (SBV)

Sofosbuvir (SBV) & Ledipasvir (LPV)
SBV and LPV show absorbance maximum at λ = 261 ± 1 & 334 ± 1 nm.
Column oven is at ambient temperature unless stated otherwise.

References:

1. Mansour, F.R., A new innovative spectrophotometric method for the simultaneous determination of sofosbuvir and ledipasvir. Spectrochimica Acta Part A: Molecular and Biomolecular Spectroscopy, 2018. 188: p. 626-632.

2. Zaman, B., F. Siddique, and W. Hassan, RP-HPLC Method for Simultaneous Determination of Sofosbuvir and Ledipasvir in Tablet Dosage Form and Its Application to In Vitro Dissolution Studies. Chromatographia, 2016. 79(23): p. 1605-1613.

3. Elkady, E.F. and A.A. Aboelwafa, A Rapid and Optimized LC-MS/MS Method for the Simultaneous Extraction and Determination of Sofosbuvir and Ledipasvir in Human Plasma. Journal of AOAC INTERNATIONAL, 2016. 99(5): p. 1252-1259.

4. Hassouna, M., M.M. Abdelrahman, and M.A. Mohamed, Assay and dissolution methods development and validation for simultaneous determination of sofosbuvir and ledipasvir by RP-HPLC method in tablet dosage forms. J Forensic Sci & Criminal Inves, 2017. 1(3): p. 001-11.

5. Saraya, R.E., M. Elhenawee, and H. Saleh, Development of a highly sensitive high-performance thin-layer chromatography method for the screening and simultaneous determination of sofosbuvir, daclatasvir, and ledipasvir in their pure forms and their different pharmaceutical formulations. Journal of Separation Science, 2018. 41(18): p. 3553-3560.

6. Abo-Talib, N.F., M.R. El-Ghobashy, and M.H. Tammam, Spectrophotometric Methods for Simultaneous Determination of Sofosbuvir and Ledipasvir (HARVONI Tablet): Comparative Study with Two Generic Products. Journal of AOAC INTERNATIONAL, 2017. 100(4): p. 976-984.

7. Khalili, M., et al., Chemometric simultaneous determination of Sofosbuvir and Ledipasvir in pharmaceutical dosage form. Spectrochimica Acta Part A: Molecular and Biomolecular Spectroscopy, 2018. 194: p. 141-151.

8. Mastanamma, S.K., et al., Development and validation of stability indicating RP-HPLC method for the simultaneous estimation of Sofosbuvir and Ledipasvir in bulk and their combined dosage form. Future Journal of Pharmaceutical Sciences, 2018. 4(2): p. 116-123.

9. El-Yazbi, A.F., et al., Eco-friendly HPTLC method for simultaneous analysis of sofosbuvir and ledipasvir in biological and pharmaceutical samples: Stability indicating study. Microchemical Journal, 2020. 154: p. 104584.

10. El-Shorbagy, H.I., et al., Optimization and modeling of a green dual detected RP-HPLC method by UV and fluorescence detectors using two level full factorial design for simultaneous determination of sofosbuvir and ledipasvir: Application to average content and uniformity of dosage unit testing. Microchemical Journal, 2019. 147: p. 374-392.

11. Mohamed, H.M., et al., Green RP-HPLC method for simultaneous determination of sofosbuvir, ledipasvir, velpatasvir antivirals and beyond in their bulk material and co-formulated products. Microchemical Journal, 2023. 186: p. 108344.

12. Rai, S.Y., Y. Prajapati, and P. Patni, Development and validation of RPHPLC and UV spectroscopy methods for simultaneous estimation of Sofosbuvir and Ledipasvir in their combined tablet dosage form. An International journal of Pharmaceutical Sciences, 2017. 8(2).

13. El-Yazbi, A.F., et al., Green analytical method for the determination of sofosbuvir, ledipasvir, ribavirin and complex silymarin flavonoids simultaneously in biological fluids. Microchemical Journal, 2021. 164: p. 105964.

14. El-Gizawy, S.M., et al., New, simple and sensitive HPTLC method for simultaneous determination of anti-hepatitis C sofosbuvir and ledipasvir in rabbit plasma. Journal of Chromatography B, 2018. 1092: p. 432-439.

15. Pan, C., et al., Simultaneous determination of ledipasvir, sofosbuvir and its metabolite in rat plasma by UPLC–MS/MS and its application to a pharmacokinetic study. Journal of Chromatography B, 2016. 1008: p. 255-259.

16. Majnooni, M.B., et al., Rapid and sensitive UHPLC-DAD method for simultaneous determination of sofosbuvir and ledipasvir in human serum. Journal of Pharmaceutical and Biomedical Analysis, 2021. 195: p. 113860.

17. Eissa, M.S., Simultaneous determination of the brand new two-drug combination for the treatment of hepatitis C: Sofosbuvir/ledipasvir using smart spectrophotometric methods manipulating ratio spectra. Spectrochimica Acta Part A: Molecular and Biomolecular Spectroscopy, 2017. 183: p. 362-370.

18. Hemdan, A. and M.S. Eissa, Simultaneous chromatographic analysis of Sofosbuvir/Ledipasvir in their combined dosage form: an application to green analytical chemistry. Journal of Analytical Science and Technology, 2019. 10(1): p. 39.

19. El-Shorbagy, H.I., et al., Earth-friendly spectrophotometric methods for simultaneous determination of ledipasvir and sofosbuvir: Application to average content and uniformity of dosage unit testing. Spectrochimica Acta Part A: Molecular and Biomolecular Spectroscopy, 2018. 205: p. 398-409.

20. Farid, N.F. and N.S. Abdelwahab, Chromatographic analysis of ledipasvir and sofosbuvir: new treatment for chronic hepatitis C infection with application to human plasma. Journal of Liquid Chromatography & Related Technologies, 2017. 40(7): p. 327-332.

21. Baker, M.M., et al., Validated spectrophotometric and chromatographic methods for analysis of the recently approved hepatitis C antiviral combination ledipasvir and sofosbuvir. Annales Pharmaceutiques Françaises, 2018. 76(1): p. 16-31.

22. Nagaraju, T., et al., A new RP-HPLC method for the simultaneous assay of sofosbuvir and ledipasvir in combined dosage form. International Journal of ChemTech Research, 2017. 10(7): p. 761-768.

23. Baker, M.M., S.F. Hammad, and T.S. Belal, Development and validation of a versatile HPLC-DAD method for simultaneous determination of the antiviral drugs daclatasvir, ledipasvir, sofosbuvir and ribavirin in presence of seven potential impurities. Application to assay of dosage forms and dissolution studies. Drug Development and Industrial Pharmacy, 2019. 45(7): p. 1111-1119.

24. Abdallah, O.M., A.M. Abdel-Megied, and A.S. Gouda, Development a validated highly sensitive LC–MS/MS method for simultaneous quantification of Ledipasvir, sofosbuvir and its major metabolite GS-331007 in human plasma: Application to a human pharmacokinetic study. Journal of Pharmaceutical and Biomedical Analysis, 2017. 143: p. 305-310.

25. Swathi, K., P.V. Rao, and N.S. Rao, A new analytical method for determination of ledipasvir and sofosbuvir in pharmaceutical formulations by HPLC method. International Journal of Research In Pharmaceutical Chemistry and Analysis, 2019. 1(3): p. 59-67.

26. Jahnavi, B., La, and S. Ganapaty, Development and Validation of a Stability-indicating Method for the Simultaneous Estimation of Sofosbuvir and Ledipasvir by RP-HPLC. Indian Journal of Pharmaceutical Sciences, 2018. 80: p. 1170-1176.

27. Kumari, K. and D. Sankar, UPLC method for simultaneous estimation of ledipasvir and sofosbuvir in bulk and dosage forms and their stress degradation studies. J Bioanal Biomed, 2019. 11(1): p. 136-141.

28. Abdelfatah, M. and M. Hassouna, Novel and facile spectrophotometric techniques for the determination of sofosbuvir and ledipasvir in their tablet dosage form. Journal of Analytical & Pharmaceutical Research, 2018. 7.

29. van Seyen, M., et al., Quantification of second generation direct-acting antivirals daclatasvir, elbasvir, grazoprevir, ledipasvir, simeprevir, sofosbuvir and velpatasvir in human plasma by UPLC-MS/MS. Journal of Chromatography B, 2019. 1110: p. 15-24.

30. Gandla, K., et al., Analytical Method Development & Validation for the Simultaneous Estimation of Ledipasvir and Sofosbuvir in Bulk and IT's Dosage Form by Rp-hplc. International Journal of Pharmaceutics and Drug Analysis, 2020. 8(4): p. 6-15.

31. Rote, A., J. Alhat, and A. Kulkarni, Development and Validation of RP-HPLC Method for the Simultaneous Estimation of Ledipasvir and Sofosbuvir in Bulk and Pharmaceutical Dosage Form. International Journal of Pharmaceutical Sciences and Drug Research, 2017. 9.

32. Trivedi, R.N., et al., Chemometrically Assisted RP-HPLC and Spectroscopic Method Development for Simultaneous Multi-Component Analysis of Ledipasvir and Sofosbuvir in Pure and Pharmaceutical Formulation. Der Pharmacia Lettre., 2018. 10: p. 62-75.

33. Mankar, S., S. Bhawar, and P. Dalavi, Development and Validation of Stability indicating RP-HPLC method for Simultaneous Estimation of Sofosbuvir and Ledipasvir in Bulk Tablet Dosage Form. Journal of Drug Delivery and Therapeutics, 2019. 9(3-s): p. 500-509.

34. Rezk, M.R., et al., Quantification of sofosbuvir and ledipasvir in human plasma by UPLC–MS/MS method: Application to fasting and fed bioequivalence studies. Journal of Chromatography B, 2016. 1028: p. 63-70.

35. Thummala, U.K., Estimation of Ledipasvir and Sofosbuvir by Vierdot's Method in Bulk and Dosage Forms. International Journal of Pharma Research, 2018. 9(1).

36. Yeram, P., P. Hamrapurkar, and P. Mukhedkar, Implementation of Quality by Design approach to develop and validate stability indicating assay method for simultaneous estimation of sofosbuvir and ledipasvir in bulk drugs and tablet formulation. Int. J. Pharm. Sci, 2019. 10: p. 180-188.

37. Ezzeldin, E., et al., Validated reversed-phase liquid chromatographic method with gradient elution for simultaneous determination of the antiviral agents: sofosbuvir, ledipasvir, daclatasvir, and simeprevir in their dosage forms. Molecules, 2020. 25(20): p. 4611.

38. Ariaudo, A., et al., A UHPLC–MS/MS method for the quantification of direct antiviral agents simeprevir, daclatasvir, ledipasvir, sofosbuvir/GS-331007, dasabuvir, ombitasvir and paritaprevir, together with ritonavir, in human plasma. Journal of Pharmaceutical and Biomedical Analysis, 2016. 125: p. 369-375.

39. Kishore, M.S. and C. Rambabu, Development and Validation of UV Spectrophotometric method for the estimation of Sofosbuvir and Ledipasvir in combined Pharmaceutical dosage forms. Int. J. ChemTech Res, 2019. 12: p. 33-40.

40. Gamal, S., et al., Simultaneous spectrophotometric determination of recombined sofosbuvir, ledipasvir and paracetamol together as commonly repurposed drugs for COVID-19 treatment. Future Journal of Pharmaceutical Sciences, 2023. 9(1): p. 71.

41. Raslan, M., et al., Simultaneous Determination of Ledipasvir/Sofosbuvir by LC/MS/MS in Human Plasma and its Pharmacokinetics Application. Saudi J Med Pharm Sci, 2022. 8(5): p. 214-226.

42. Salama, F.M., et al., Different spectral data processing techniques for determination of ledipasvir and sofosbuvir in their pure and dosage forms; a comparative study. International Journal of Science, 2018. 5(1): p. 16-21.

43. Gamal, S., et al., Rapid and validated UHPLC method for simultaneous determination of sofosbuvir, ledipasvir and paracetamol as commonly repurposed drugs for COVID-19 treatment: application in spiked human plasma. Future Journal of Pharmaceutical Sciences, 2023. 9(1): p. 92.

44. Labidi, A. and L.L. El Atrache, Chemometrically Assisted Development of Ultra-High-Performance Liquid Chromatography Method for the Simultaneous Quantification of Sofosbuvir, Daclatasvir and Ledipasvir in Pharmaceutical Dosage Forms. Journal of Chromatographic Science, 2019. 57(10): p. 910-919.

45. El-Waey, A.A., et al., Eco friendly stability indicating HPTLC method for simultaneous determination of sofosbuvir and ledipasvir in pharmaceutical tablets and HPTLC-MS characterization of their degradation products. Microchemical Journal, 2023. 186: p. 108324.

46. El-Shorbagy, H.I., et al., A green stability-indicating RP-HPLC-UV method using factorial design for determination of ribavirin, sofosbuvir and ledipasvir: Application to average content, acid degradation kinetics and in vitro drug interactions study. Microchemical Journal, 2020. 158: p. 105251.

47. Surya, R.P.S.B.R. and M.S.M.M.S. Maduri, Stability indicating method development and validation for the simultaneous estimation of ledipasvir and sofosbuvir in bulk drug by using RP-HPLC. World Journal of Current Medical and Pharmaceutical Research, 2020: p. 307-318.

48. Jayaprakash, R., et al., ANALYTICAL METHOD DEVELOPMENT AND VALIDATION FOR THE ESTIMATION OF SOFOSBUVIR AND LEDIPASVIR IN RAW MATERIAL AND TABLET FORMULATION BY HIGH PERFORMANCE THIN LAYER CHROMATOGRAPHIC (HPTLC) METHOD. 2021.

49. Kumar, D.V. and J. Rao, A new validated stability indicating rp-hplc method for simutaneous estimation of sofosbuvir and ledipasvir in tablet dosage forms. World J Pharm Res, 2018. 7: p. 763-78.

50. Mujawar, T., et al., Development and validation for the simultaneous estimation of Sofosbuvir and Ledipasvir by UV spectrophotometer method in bulk and tablet dosage forms.

51. Nasr, J.J. and S. Shalan, Validated 1H and 19F nuclear magnetic resonance for the quantitative determination of the hepatitis C antiviral drugs sofosbuvir, ledipasvir, and daclatasvir in tablet dosage forms. Microchemical Journal, 2020. 152: p. 104437.

52. Sunder, B.S. and A.K. Mittal, Bio-analytical method development and validation for simultaneous determination of ledipasvir and sofosbuvir drugs in human plasma by RP-HPLC method. Int J Curr Pharm Res, 2018. 10(3): p. 21-26.

53. Bhavani, R.P.S. and M.S. Maduri, Stability indicating method development and validation for the simultaneous estimation of ledipasvir and sofosbuvir in bulk drug by using RP-HPLC. World Journal of Current Medical and Pharmaceutical Research, 2020. 2.

54. Salama, F., et al., Multivariate chemometric models and application of genetic algorithm for simultaneous determination of ledipasvir and sofosbuvir in pure form and in pharmaceutical preparation; a comparative study. Journal of Advanced Pharmacy Research, 2017. 1(4): p. 185-192.

55. Salama, F.M., et al., Application of TLC densitometric method for simultaneous estimation of the newly co-formulated antiviral agents ledipasvir and sofosbuvir in their tablet dosage form. Analytical Chemistry Letters, 2017. 7(2): p. 241-247.

56. Satheshkumar, S., Development of Validated UV Spectroscopic, HPTLC and RP-HPLC Methods for the Simultaneous Estimation of Ledipasvir and Sofosbuvir in Pure and Fixed Dose Combination. 2017, Sri Ramakrishna Institute of Paramedical Sciences, Coimbatore.

57. Moustafa, H., et al., Economic and Green RP-HPLC Method for Simultaneous Determination of Sofosbuvir, Ledipasvir, Velpatasvir Antivirals and Beyond in Their Bulk Material and Co-Formulated Products. Ledipasvir, Velpatasvir Antivirals and Beyond in Their Bulk Material and Co-Formulated Products.

58. Aboras, S.I., et al., In-depth investigation of the Silymarin effect on the pharmacokinetic parameters of sofosbuvir, GS-331007 and ledipasvir in rat plasma using LC–MS. Biomedical Chromatography, 2022. 36(9): p. e5427.

59. Safakhoo, N., et al., Enhancement spectral resolution for the prediction amount of sofosbuvir and ledipasvir using least squares support vector machine and artificial neural networks in pharmaceutical formulation. Journal of the Chilean Chemical Society, 2019. 64(1): p. 4310-4323.

60. Bhangale, C.J. and S.N. Hiremath, QUANTIFICATION OF SOFOSBUVIR AND LEDIPASVIR IN BULK AND DOSAGE FORM BY HPTLC AND RP-HPLC METHODS IN PRESENCE OF ITS DEGRADATION PRODUCTS.

61. Mabrouk, M., I.I. Abdelfattah, and F.R. Mansour, Green method for determination of four anti-viral drugs using micellar liquid chromatography: Application to dosage form analysis. Sustainable Chemistry and Pharmacy, 2023. 35: p. 101202.

62. Abdulkareem, A. and M.F. El-Tohamy, Validated capillary zone electrophoresis approach for simultaneous separation and determination of hepatitis C Sofosbuvir and Ledipasvir in tablet dosage form. World J Pharm Res, 2017. 6: p. 129-147.

63. Rageh, A.H., F.A.M. Abdel-aal, and U. Pyell, Optimization of a sensitive and robust strategy for micellar electrokinetic chromatographic analysis of sofosbuvir in combination with its co-formulated hepatitis C antiviral drugs. Journal of Chromatography A, 2020. 1616: p. 460795.

64. Elkhoudary, M.M., et al., Development and validation of a simple HPTLC method for the determination of new hepatitis C subtype 4 antiviral agents in their tablet dosage form. JPC–Journal of Planar Chromatography–Modern TLC, 2020. 33: p. 71-77.

65. Atta, N.F., A. Galal, and Y.M. Ahmed, New strategy for determination of anti-viral drugs based on highly conductive layered composite of MnO2/graphene/ionic liquid crystal/carbon nanotubes. Journal of Electroanalytical Chemistry, 2019. 838: p. 107-118.

66. Atta, N.F., A. Galal, and Y.M. Ahmed, Electrochemical method for the determination of three new anti-hepatitis C drugs: application in human blood serum. Journal of The Electrochemical Society, 2018. 165(10): p. B442.

67. Ibrahim, A.E., et al., Comparison between core-shell and totally porous particle stationary phases for fast and green LC determination of five hepatitis-C antiviral drugs. J Sep Sci, 2018. 41(8): p. 1734-1742.

68. Kannouma, R.E., et al., A dispersive liquid–liquid microextraction method based on solidification of floating organic droplet for determination of antiviral agents in environmental water using HPLC/UV. Microchemical Journal, 2021. 171: p. 106790.

69. Bendas, E.R., M.R. Rezk, and K.A. Badr, Drug Interchangeability of Generic and Brand Products of Fixed Dose Combination Tablets of Sofosbuvir and Ledipasvir (400/90 mg): Employment of Reference Scaled Average Bioequivalence Study on Healthy Egyptian Volunteers. Clinical Drug Investigation, 2018. 38(5): p. 439-448.

70. El-Yazbi, A.F., et al., Cost-effective green chromatographic method for the simultaneous determination of four commonly used direct-acting antiviral drugs in plasma and various pharmaceutical formulations. Microchemical Journal, 2021. 168: p. 106512.

71. Shaik Karishma, P.K., B. Sivagami, M. Niranjan Babu, Narayanaswamy Harikrishnan, Rp-Hplc Method Development And Validation For The Simultaneous Estimation Of Ledipasvir And Sofosbuvir In Fixed Dosage Form Journal of Global Trends in Pharmaceutical Sciences, 2021. 12: p. 8928 - 8934.

72. K. Yogendrachari, M.M., E. Gireesh Kumar, M.Vasantha kumari, M. Chanti Naik Analytical method development and validation for simultaneous determination of ledipasvir and sofosbuvir in tablet dosage form by rp-hplc Journal of Global Trends in Pharmaceutical Sciences, 2016. 7: p. 3401- 3407

73. Jampana, R.T., P.R. Avula, and P.D. Anumolu, Multivariate optimization and evaluation of quaternary mixture in bulk and co-formulated dosage forms by central composite design. Future Journal of Pharmaceutical Sciences, 2021. 7(1): p. 111.

74. Ismail, Y. and M. VijayaVaraPrasad, Simultaneous estimation of ledipasvir and sofosbuvir in bulk and its dosage forms by stability indicating RP-HPLC method. International Journal of Research in Pharmaceutical Sciences, 2019.

75. Evangelin, M., et al., DEVELOPMENT AND VALIDATION OF STABILITY INDICATING RP-HPLC METHOD FOR SIMULTANEOUS ESTIMATION OF SOFOSBUVIR AND LEDIPASVIR IN TABLET DOSAGE FORM. 2019. 4: p. 4815-4821.

76. A, S., S. S, and R. T.K, "Development of validated specific stability-indicating HPTLC method for the simultaneous determination of Ledipasvir and Sofosbuvir in fixed dose tablet formulation". Asian Journal of Nanoscience and Materials, 2019. 2(2): p. 228-243.

77. Sathiya, C. and S. Badeliya, Analytical method development and validation for simultaneous estimation of Sofosbuvir and Ledipasvir in tablet dosage form. World Journal of Pharmacy and Pharmaceutical Sciences, 2018. 7(7): p. 897-914.

78. Rao, B.S., M. Reddy, and B. Rao, Simultaneous analysis of ledipasvir and sofosbuvir in bulk and tablet dosage form by stability indicating high performance liquid chromatographic method. Global Journal for Research Analysis, 2017. 6(4): p. 505-509.

79. Kranthi Kiran, K., et al., A new analytical method development and validation for the simultaneous estimation of ledipasvir and sofosbuvir using RP-HPLC. ICJPIR, 2017. 4(1): p. 142-165.

80. Abo-Zeid, M.N., et al., Ultrasensitive spectrofluorimetric method for rapid determination of daclatasvir and ledipasvir in human plasma and pharmaceutical formulations. Journal of Pharmaceutical and Biomedical Analysis, 2018. 152: p. 155-164.

81. Abdel-Lateef, M.A., et al., Micellar-based spectrofluorimetric method for the selective determination of ledipasvir in the presence of sofosbuvir: application to dosage forms and human plasma. Luminescence, 2020. 35(4): p. 486-492.

82. Salama, F.M., et al., Spectroflurimetric estimation of the new antiviral agent ledipasvir in presence of sofosbuvir. Spectrochimica Acta Part A: Molecular and Biomolecular Spectroscopy, 2018. 190: p. 513-517.

83. Hamad, A.E., et al., Micelle sensitized synchronous spectrofluorimetric approaches for the simultaneous determination of simeprevir and ledipasvir: Application to pharmaceutical formulations and human plasma. Spectrochimica Acta Part A: Molecular and Biomolecular Spectroscopy, 2020. 239: p. 118471.

84. Ali, R., et al., Specific stability indicating spectrofluorimetric method for determination of ledipasvir in the presence of its confirmed degradation products; application in human plasma. Spectrochimica Acta Part A: Molecular and Biomolecular Spectroscopy, 2018. 202: p. 50-57.

85. Abdel-Lateef, M.A., et al., Novel spectrofluorimetric approach for determination of ledipasvir through UV-irradiation: application to biological fluids, pharmacokinetic study and content uniformity test. RSC advances, 2019. 9(59): p. 34256-34264.

86. Atty, S.A., et al., Graphite/nanocrystalline zeolite platform for selective electrochemical determination of hepatitis C inhibitor ledipasvir. Electroanalysis, 2019. 31(7): p. 1215-1223.

87. Allahverdiyeva, S., et al., Electroanalytical investigation and determination of hepatitis C antiviral drug ledipasvir at a non-modified boron-doped diamond electrode. Diamond and Related Materials, 2020. 108: p. 107962.

88. Abdel-aal, F.A.M., et al., ε-MnO2-modified graphite electrode as a novel electrochemical sensor for the ultrasensitive detection of the newly FDA approved Hepatitis C antiviral drug ledipasvir. Analytica Chimica Acta, 2018. 1038: p. 29-40.

89. Tawab, M.A.H.A., M.G.A. El-Moghny, and R.M. El Nashar, Computational design of molecularly imprinted polymer for electrochemical sensing and stability indicating study of sofosbuvir. Microchemical Journal, 2020. 158: p. 105180.

90. El-Wekil, M.M., et al., A facile synthesis of 3D NiFe2O4 nanospheres anchored on a novel ionic liquid modified reduced graphene oxide for electrochemical sensing of ledipasvir: Application to human pharmacokinetic study. Biosensors and Bioelectronics, 2018. 109: p. 164-170.

91. Mahmoud, A.M., et al., Modification of N,S co-doped graphene quantum dots with p-aminothiophenol-functionalized gold nanoparticles for molecular imprint-based voltammetric determination of the antiviral drug sofosbuvir. Microchimica Acta, 2019. 186(9): p. 617.

92. Soliman, M.A., et al., Electrochemical sensor based on bio-inspired molecularly imprinted polymer for sofosbuvir detection. RSC Adv, 2023. 13(36): p. 25129-25139.
